# Coupling UiO-66 MOF with a Nanotubular Oxide Layer Grown on Ti-W Alloy Accelerates the Degradation of Hormones in Real Water Matrices

**DOI:** 10.1021/acsomega.4c07470

**Published:** 2024-11-22

**Authors:** Isabela Disigant, Juliana de Almeida, Débora Noma Okamoto, Rodnei Bertazzoli, Christiane de Arruda Rodrigues

**Affiliations:** † Department of Chemical Engineering, Instituto de Ciências Ambientais, Químicas Farmacêuticas, Universidade Federal de São Paulo, Rua São Nicolau, 210, Diadema, Sao Paulo 09913-030, Brazil; ‡ Unesp, National Institute for Alternative Technologies of Detection, Toxicological Evaluation and Removal of Micropollutants and Radioactives (INCT-DATREM), Institute of Chemistry, P.O. Box 355, Araraquara, Sao Paulo 14800-900, Brazil; § Department of Pharmaceutical Science, Instituto de Ciências Ambientais, Químicas Farmacêuticas, Universidade Federal de São Paulo, Rua São Nicolau, 210, Diadema, Sao Paulo 09913-030, Brazil; ∥ School of Mechanical Engineering, Universidade Estadual de Campinas, Rua Mendeleyev, 200, Campinas, Sao Paulo 13083-860, Brazil

## Abstract

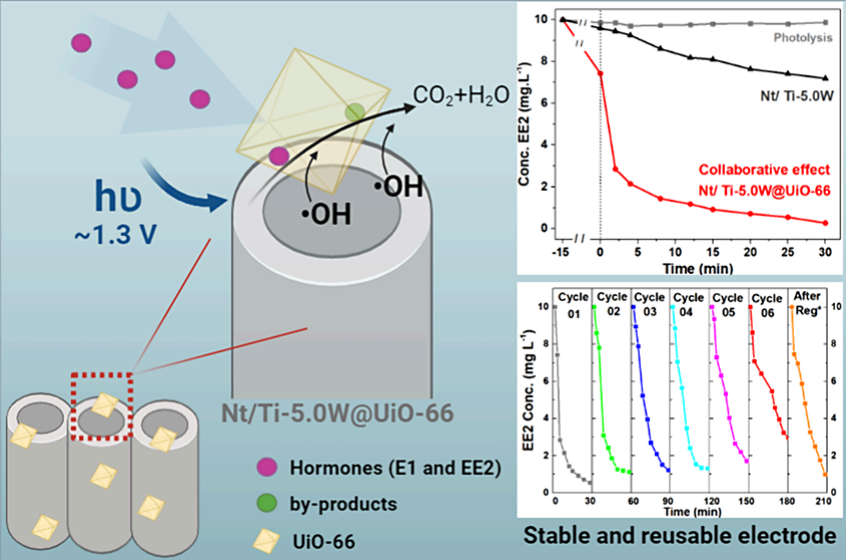

To enable the photoelectrocatalytic treatment of large volumes of water containing low concentrations of pollutants, this study introduces a hybrid photocatalyst, composed of nanotubular oxides grown on Ti*x*W alloy (*x* = 0.5 and 5.0 wt %) modified with UiO-66 MOF, for degradation of estrone (E1) and 17α-ethinyl estradiol (EE2). The oxide layer (Nt/Ti*x*W) was prepared via anodization, while UiO-66 nanoparticles were synthesized by using a solvothermal process. Different techniques for modifying nanotubular oxides were evaluated to maximize the photocatalytic activity and the sorption process. In photo(electro)catalytic experiments using low concentrations of E1 and EE2 synthetic solutions and UV–vis radiation (100 W/cm^2^), all modified materials exhibited approximately 40% higher degradation compared to the unmodified photocatalyst, keeping the same sequential performance of the photocatalysts (Nt/TiO_2_ < Nt/Ti-0.5W < Nt/Ti-5.0W) independent of the treatment. This enhancement was attributed to the MOF’s increased hormone sorption, with no synergistic interaction observed between the photocatalyst and the adsorbent. In real water supply matrices, the photoelectrocatalytic removal rate of E1 using Nt/Ti-5.0W modified UiO-66 under UV–vis radiation and 1.3 V was 0.168 s^–1^, while for EE2, it was 0.310 min^–1^, approximately 1.78 and 18.21 times faster than obtained with the unmodified photocatalyst. The slower degradation rate of EE2 compared to that of E1 is attributed to the formation of denser intermediates that compete with smaller organic molecules in the real matrix. The cooperative effect between NT/Ti*x*W and UiO-66 favored the confinement of pollutants and by-products within the UiO-66 cavity, minimizing the diffusion effects and promoting the degradation of these compounds by the OH^·^ radical generated at the oxide/solution interface. Among the tested electrodes, NT/Ti5W modified with UiO-66 demonstrated the highest efficiency and stability during the recycle tests. This highlights its promise for applications in photocatalytic processes for treating water supplies with low pollutant concentrations.

## 1 Introduction

Emerging contaminants appear in water supplies at low concentrations (μg L^–1^ and ng L^–1^), presenting challenges in their detection, removal, and degradation.^1^ This category covers diverse chemical compounds of natural or synthetic origin, including exogenous substances, such as estrone (E1) and 17α-ethinyl estradiol (EE2). The presence of hormones E1 and EE2 in water sources can harm the environment, ecosystem, and human health, causing toxicity and even carcinogenicity.^2−4^ E1, a naturally occurring hormone, arises either endogenously within the body or through the degradation of 17β-estradiol.^3,5^ In contrast, EE2 stands out as a principal hormonal constituent employed in the formulation of oral contraceptives, ranking among the most widely consumed pharmaceuticals globally.^3^ The introduction of these hormones into water bodies occurs through nonmetabolized excretion and the application of natural fertilizers,^6−8^ resulting in their presence in supply and wastewater at exceedingly low concentrations. While they have been widely implemented, conventional drinking water and sewage treatment processes have proven inadequate in eliminating these compounds. As a result, research efforts^6,9−11^ have focused on developing economically viable, efficient, and rapid treatment methods for supplying water to facilitate the large-scale removal of these substances. This is particularly crucial in contexts characterized by significant water volumes and rapid flows, such as municipal supply water treatment plants.

Among these techniques, photocatalytic and/or photoelectrocatalytic oxidation are considered alternative processes for removing organic loads and eliminating organic pollutants and recalcitrant products.^12,13^ The operation of this technique is directly related to the semiconductor used as a catalyst in the process, which promotes the migration of an electron from the valence band to the conduction band from light irradiation and, consequently, photogeneration of electrons and holes, which will promote the degradation of the organic compound directly or indirectly.^13^ Nevertheless, the holes and electrons generated have a short lifetime, reducing the production of hydroxyl radicals and/or their availability for interaction with the exposed organic compound.^14,15^ Furthermore, some semiconductors have an unfavorable valence band or conduction band position for generating hydroxyl radicals (^·^OH) and the production of superoxide anion radicals (O_2_^·–^) from the oxygen reduction, respectively. To overcome these barriers, semiconductors are modified with metals or metal oxides,^15^ resulting in a decrease in the recombination rates of electron/hole pairs, improvement of charge transfer at the semiconductor/solution interface, and the use of visible light.^15^

Semiconductors based on titanium dioxide (TiO_2_) are widely used due to their high oxidation capacity under ultraviolet light, chemical stability, and quantum yield.^16−19^ However, TiO_2_ has a high rate of recombination of electron–hole pairs and excitation only under UV radiation, which increases the energy consumption of the process. Recent studies have shown that modifying TiO_2_ with oxides of tungsten (W), niobium (Nb), and copper (Cu) can reduce these deficiencies, enhancing the efficiency of photocatalytic and photoelectrocatalytic processes.^6,9,20^ According to de Almeida, the EE2 concentration in an enriched real matrix was reduced by ∼77% after 8 h using visible light with a TiO_2_ electrode doped with 5.0 wt % W. In another study, Kaur et al. used photoelectrocatalysis for municipal wastewater disinfection employing a TiO_2_ catalyst modified with tin oxide and mesoporous carbon (TiO_2_-G-SnO_2_). They achieved a 70% removal of organic load after 180 min under visible light radiation at 400 nm, representing a 20% increase compared to treatment using only UVC irradiation during the same period.^21^

Another limitation of photoelectrochemical systems is the diffusion process of species onto the electrode surface. The low concentration of the pollutant in aqueous media can hinder the diffusion rate; however, this can be improved by implementing a preconcentration step for the pollutant before the treatment.^22^ In this context, three-dimensional porous structures composed of metallic ions or clusters connected to organic molecules, known as metal–organic frameworks (MOFs), have an enhanced pollutant removal efficiency. MOFs exhibit a high absorption capacity and can improve the removal of pollutants at low concentrations owing to their large surface area, pore size, and morphology.^23^ Consequently, these materials have demonstrated effectiveness in heterogeneous catalysis processes, improving the efficiency and reaction selectivity. The porous structure of MOFs contains active sites for organic and inorganic compounds, making them a valuable and efficient alternative for heterogeneous catalysis. Compared to other adsorbent materials, MOFs offer the advantage of stable pores with specific and uniform sizes.^24^

Overall, metal–organic structures are considered promising adsorbent materials in water treatment, effectively removing and preconcentrating emerging contaminants. These structures are often combined with other processes, such as photocatalytic oxidation/reduction or membrane filtration, to enhance treatment efficiency.^25,26^ Therefore, chemical stability and insolubility in water are crucial factors determining the practical applicability of MOFs in water treatment processes,^27,28^ and these properties can be easily compromised due to the attack of the metal–organic coordination bonds by water molecules. Crystallinity, metal–ligand coordination geometry, central metal ions, porosity, organic ligands, and pore surface are among the characteristics that affect the stability of the material.^27,29,30^ Specific combinations of these characteristics can prevent the breakage or displacement of metal–organic ligands or change the crystal phases and, consequently, avoid the collapse of MOF structures.^27,28,30^

Among many reported MOFs, only a few exhibit stability in aqueous media, such as Zr-based MOFs, especially UiO-66.^29,31,32^ The stability of UiO-66 in aqueous applications is due to the strong Zr–O bond and a specific geometry that prevents water molecules from entering in the pores, minimizing hydrolysis reactions and preserving its crystalline structure even after water exposure.^32,33^ In addition, it has a highly porous structure with a large specific surface area, usually greater than 1000 m^2^/g, which provides the material with active sites that contribute to adsorption and interaction with contaminants.^27^

Therefore, MOF-UiO-66 has garnered significant attention in effluent treatment, particularly in the removal of textile dyes,^34^ pharmaceuticals,^21,35^ and ion metals.^36^ UiO-66 MOF has also been used in photocatalytic processes. In a study by Melillo et al., UiO-66 was combined with other metals, including Zr, Ce, and Ti. The mixed-metal UiO-66 demonstrates higher catalytic efficiency, regardless of the metal species studied, compared to pure UiO-66, achieving a catalytic response exceeding 40% in the UV region.^37^ In another research, Han et al. synthesized the UiO-66/MoSe_2_ composite and evaluated its photocatalytic properties in the degradation rate of Rhodamine B and the reduction of Cr(VI). The RhB wastewater was completely decolorized within 120 min, and most of the Cr(VI) was reduced after 150 min.^38^ Compared to the two pure catalysts, the composite exhibited significantly improved photocatalytic degradation of wastewater containing RhB and reduced Cr(VI).^38^

When analyzing the UiO-66 MOF application in water treatment, its stability in aqueous environments, as well as its thermal and chemical stability, can be highlighted, which allows it to be used in aggressive environments, and its large surface area and porosity maximize its ability to adsorb pollutants. In addition, UiO-66 can be easily functionalized, allowing adjustments to be made to its surface properties to optimize the adsorption and degradation of specific contaminants.^27^ On the other hand, UiO-66 has some disadvantages that should be considered. One of these is its susceptibility to degradation under extremely basic pH conditions (greater than 13), which may limit its application in certain industrial environments.^27^ In addition, large-scale synthesis can be a challenge, as the crystalline nature of UiO-66 requires specific production methods, which can be costly and complex.^27^ Despite these limitations, UiO-66 remains a promising choice due to its versatility and performance in adverse conditions, especially its stability in aqueous environments, which makes it a robust choice for water treatment applications.

Motivated by these promising results and considering various interactions of UiO-66 with emerging pollutants, the hybrid assembly composed of UiO-66 MOFs and Ti-W oxide nanotubes, a relatively unexplored combination, appears as a potential way to enhance the performance of photocatalysts for removing hormones from supply water.

In this context, a nanotube oxide layer grown on pure Ti (Nt/TiO_2_) and on Ti-*x*W alloys, where *x* = 0.5 and 5.0% by weight (Nt/Ti-*x*W), was prepared by anodization and subsequently modified with UiO-66 nanoparticles via solvothermal synthesis. The UiO-66 was chosen as a modifier agent due to its remarkable stability in aqueous environments and high capacity of functionalization, allowing adjustments to the surface properties to optimize the adsorption and degradation of specific contaminants. The performance of these materials was evaluated in the photocatalytic degradation of hormones E1 and EE2 in real matrices. The choice of alloy composition was based on previous research (de Almeida et al.), which explored two modification processes of TiO_2_ nanotubes with W: one involving doping (Nt/Ti-0.5W) and another involving doping and heterojunction (Nt/Ti-5.0W).^6^ In the latter, a semiconductor with a low recombination rate and UV and visible light absorptions is obtained. The influence of the UiO-66 deposition amount on the nanotube oxide layer was studied to optimize the operational conditions of the photocatalyst, considering both photocatalytic activity and the adsorption process. Degradation studies were conducted using these photocatalysts with and without UiO-66, under various photo(electro)chemical conditions, and using real water supply matrices. The stability and reusability of the materials were evaluated, and a proposed photocatalytic activation mechanism associated with the adsorption process was presented.

## 2 Materials and Methods

### 2.1 Synthesis and Characterization of UiO-66 MOF

**Figure 1:**
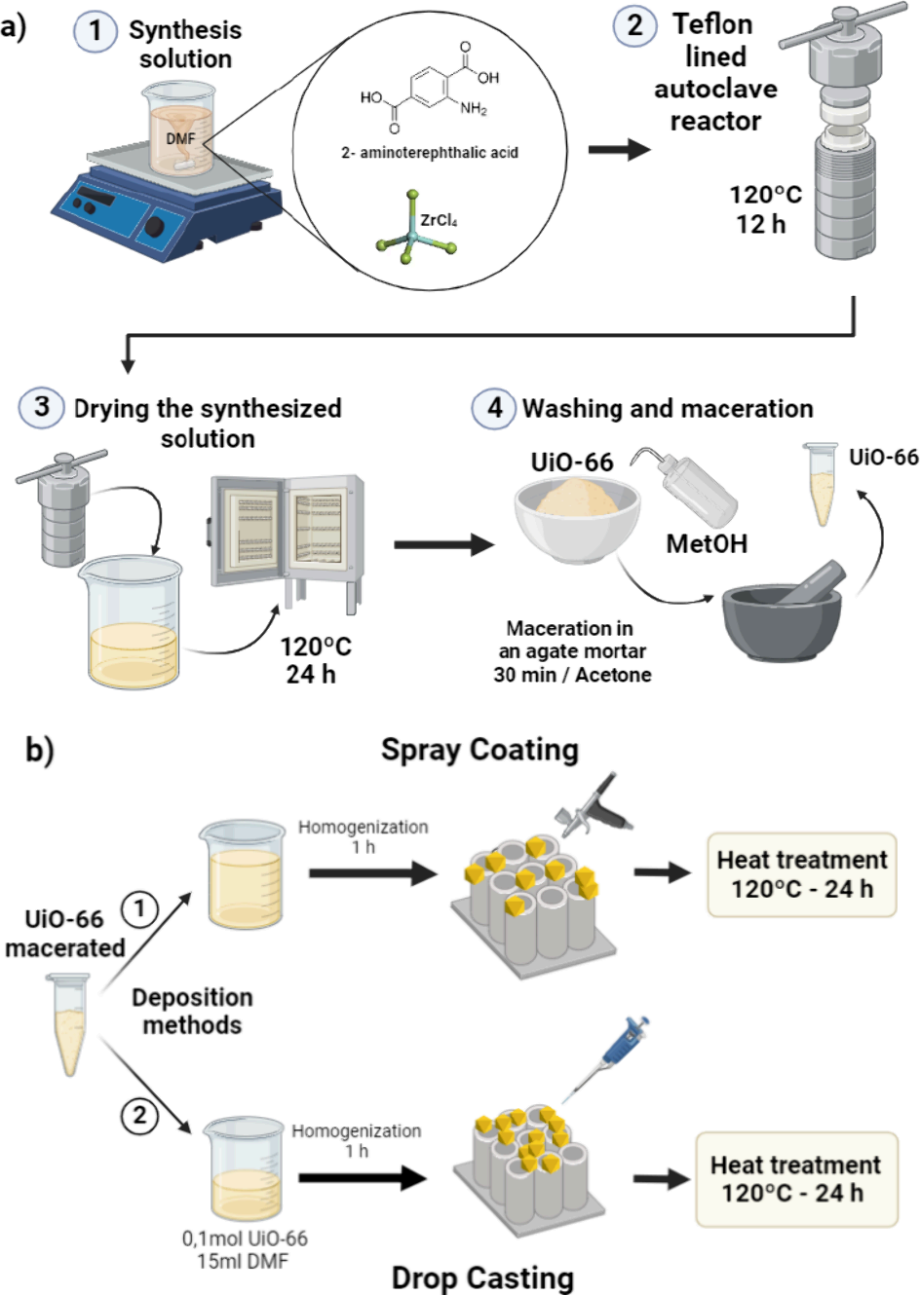
Schematic illustration of (a) the synthesis process of UiO-66 MOF by the solvothermal method and (b) the modification of nanotubular oxide electrodes with UiO-66 nanoparticles using spray-coating (step 1) and drop-casting (step 2).

UiO-66 MOF was synthesized in a Teflon-lined stainless-steel autoclave, using an optimized solution containing 23.0 mg of ZrCl_4_· (zirconium chloride, >99.5%, Sigma-Aldrich), 18.0 mg of H_2_BDC-NH_2_ (2-amino terephthalic acid, 99%, Sigma-Aldrich), 6.0 μL of CH_3_COOH (acetic acid, 99%, Synth), and 5.0 mL of DMF (*N*,*N*-dimethylformamide, 99.8%, Sinergia Cientifica), after stirring for 1 h. The autoclave reactor was maintained at 120 °C for 12 h in an oven with a digital timer and then cooled to room temperature. After the reaction, the powder was thoroughly washed using a DMF solvent and dried at 120 °C for 12 h to remove the remaining organic traces. As-prepared UiO-66 were macerated in an agate mortar and pestle and suspended in DMF. Subsequently, the suspension was slightly poured onto the nanotubes from a distance of 6.0 cm. Figure 1a illustrates the schematic representation of the synthesis of the MOF UiO-66 (Zr) by the solvothermal method followed by maceration of the material.

The crystalline phase and water stability of as-prepared UiO-66 were analyzed by XRD data. Diffraction data were collected at room temperature with an angular range of 2θ = 5–60° and a step size of Δ2θ = 0.020° (Bruker, Advance D8) using Cu Kα radiation and a Bragg–Brentano optical setup. The lamellar preparation for transmission electron microscopy (TEM) analysis was prepared using SEM with focused ion beam (SEM-FIB) (Thermo Scientific Dual-Beam system) in which the ion beam and the electron intersect each other at a 52° angle near the sample surface. The samples were first coated with a thick layer of Pt (30 nm), and the lamella was cut with the assistance of the focused gallium ion beam to visualize the cross section. The bright-field transmission scanning electron microscopy (BF-STEM) images of UiO-66 nanoparticles were recorded by SEM-FIB, and the TEM images were analyzed in a FEI TECNAI G^2^ F20 HRTEM.

The thermal stability of the as-synthesized UiO-66 nanoparticles was evaluated by thermogravimetric analysis (DSC/TGA, SDT 650, TA). Brunauer–Emmett–Teller (BET) analysis and the Barrett–Joyner–Halenda method were used to verify the specific surface area, pores, and cavities of the MOF from the nitrogen adsorption–desorption isotherm connected with an isotope ratio mass spectrometer (Flash EA 1112—Thermo Scientific). Meanwhile, Fourier transform infrared (FTIR) vibrational spectra were obtained from 4000 to 400 cm^–1^ (Shimadzu, IR Prestige-21).

X-ray photoelectron spectroscopy (XPS) spectra were recorded using a K-alpha Thermo Scientific spectrometer with a monochromatic Al Kα X-ray source (*h*ν = 1486.6 eV) operating at 150 W under pressure fixed to 4.8 × 10^–9^ mbar. Spectra were charge-corrected to the main line of the C 1s (aromatic carbon) set to 284.7 eV and deconvoluted by OriginPro software using linear background subtraction and fitted with a Gaussian function. The high-resolution XPS analyses were carried out only in the UiO-66 MOF element region. These results were shown and discussed in a previous work^6^ for element spectra from Ti–O–W oxide.

### 2.2 Nanotubular Oxide Layer Modified with UiO-66 Nanoparticles

First, Ti, Ti-0.5W (wt %), and Ti-5.0W (wt %) alloys were prepared using titanium foil (99.9%—Ti-Brasil) and tungsten powder (99.9%—Aldrich, 12 μm), which were arc-melted in an argon atmosphere, followed by lamination until 2 mm thick and cut into samples measuring 30 mm × 50 mm (length × width). The substrate characterization of Ti-0.5W (wt %) and Ti-5.0W (wt %) alloys was described by de Almeida et al.^6^

After that, the nanotube layers were prepared by anodization using a two-electrode cell setup with Ti-*x*W (0 ≤ *x* ≤ 5 wt %) alloy as the working electrode and a platinum foil as the counter electrode, using an electrolyte containing 0.2 mol L^–1^ HF (48%, MERCK) + 7.5 vol % H_2_O dissolved in ethylene glycol (MERCK). The potential at 120 V was maintained constant for 30 and 40 min for Ti-0.5W (wt %) and Ti-5.0W (wt %), respectively. The condition for preparation of Ti pure was modified to reach nanotubes with similar dimensions as grown on Ti-*x*W alloy. Then, Ti pure was anodized at 20 V for 6 h using a programmable power source (Supplier, DC). After anodization, nanotubular array layers grown on Ti sheet and Ti-0.5W (wt %) were annealed at 450 °C, while the nanotubes obtained on Ti-5.0W (wt %) alloy were heat-treated at 550 °C for 150 min in atmospheric air using a muffle furnace (EDG, F3000). A UiO-66 MOF dissolved in DMF was deposited directly onto the Nt/TiO_2_ and Nt/Ti-*x*W (*x* = 0.5 and 5.0 wt %) layers.

Previously, two different methods of MOF deposition were investigated using TiO_2_ nanotubes: drop-casting (Figure 1b—step 1) and spray-coating (Figure 1b—step 2). Nt/TiO_2_ decorated with UiO-66 nanoparticles were evaluated by photocatalytic activity to identify the better deposition method. Photoelectrochemical measurements were performed by linear scan voltammetry in a single-compartment electrochemical cell configured in a conventional setup of a three-electrode with nanotube layer modified with UiO-66 as the working electrode, platinum spiral as the counter electrode, and Ag/AgCl/3 M KCl as the reference electrode, conducted by a potentiostat/galvanostat equipment (Autolab Metrohm—PGSTAT302). The electrodes were immersed in 0.2 mol L^–1^ Na_2_SO_4_, pH 5.7 as the electrolyte, with the exposed area of the working electrode being 0.282 cm^2^ under UV–vis irradiation (100 mW/cm^2^).

### 2.3 Hormone Degradation Experiments

The degradation experiments of E1 and EE2 (photolytic, photocatalytic, and photoelectrocatalytic processes) were carried out in a borosilicate glass reactor with a single compartment and equipped with a water jacket to control the reaction temperature. The Nt/TiO_2_ or Nt/Ti-*x*W@UiO-66 electrodes were placed parallel inside the reactor, and a platinum mesh was used as a counter electrode during photocatalytic experiments. When necessary, the electrodes were connected to a power supply (MCE power supplier, 1650), and a bias potential of 1.3 V was applied to the anodes. A Hg lamp (80 W) was used as a UV–vis light source, irradiating the electrode surface at 100 mW cm^–2^.

The photoelectrocatalytic degradation was evaluated using a synthesized water (pH 7—Table S1) and real water matrix (pH 7.5) containing E1 or EE2 (10 mg L^–1^), dissolved in 40 mL of H_2_O. The real water matrix samples were collected at the entrance of ETA Taiçubepa (latitude: 23°56′99.6″S, longitude: 46°29′55.8″W), in Suzano, Brazil (metropolitan region of São Paulo), to which were added aliquots of hormones E1 and EE2. The water parameters were measured by the local drinking water provider such as temperature (23 °C), pH 7.5, electric conductivity (213 μS cm^–1^), dissolved oxygen (9.05 mg L^–1^), total particulate (1731 μg L^–1^), chlorophyll (9.3 mg L^–1^), NO_2_ (1623 μg L^–1^), NH_4_ (6098 μg L^–1^), NO_3_ (2540 μg L^–1^), and total nitrogen (17,694 μg L^–1^). The presence of hormones (E1 and EE2 ) was not observed in the real water matrix, probably because the concentrations of these hormones were below the lower limit of detection of the HPLC technique, as values reported by Oliveira et al. (2020).^9^

To ensure the adsorption–desorption equilibrium, the concentration of the pollutant in the real water matrix was monitored by measuring the peak of the chromatographic spectrum after contact with Nt/Ti-*x*W for 15 min in the dark. Solution aliquots of 750 μm were taken out at regular intervals.

After degradation experiments, the compositional stability of electrodes and by-product adsorption were analyzed using X-ray photoemission spectroscopy on Nt/Ti-5.0W@UiO-66 after the photoelectrocatalytic process for EE2 removal added in a real water matrix. The analysis used the same setup and conditions described in Section 2.2. To evaluate the photocatalytic reusability of Nt/Ti-5.0W@UiO-66, the recycling photoelectrocatalytic experiments toward the degradation of EE2 in matrix water were driven under the same conditions as degradation experiments for the real water matrix enriched with hormone.

After the sixth cycle of the photoelectrocatalytic experiment, the photocatalyst was recovered by an extended photoelectrocatalytic process without a pollutant and reused for subsequent reaction. Photoelectrochemical electrode stability tests employed chronoamperometric measurements at a fixed potential (1.0 eV vs Ag/AgCl) for 2 h using a 0.1 M borate buffer (pH 7.2) solution as the electrolyte. The UV/vis irradiation (100 mW/cm^2^) was kept constant.

### 2.4 Antibacterial and Toxicity Assays

In UiO-66, the experiments were conducted using two methods: the disk diffusion method and the broth liquid method using MTT (3-(4,5-dimethylthiazol-2-yl)-2,5-diphenyltetrazolium bromide). Disk diffusion methods measure the bacterial growth inhibition zone around paper disks impregnated with a specific target concentration on agar plates. In this case, we adapted the method because the sample is a powder. The disk was made using UiO-66 as the substrate. Bacterial inoculum (*Escherichia coli* and *Staphylococcus aureus*) was prepared by suspending colonies in a Luria–Bertani (LB) medium to the density (λ = 600 nm) of 0.1 (approximately 108 CFU/mL). The inoculum was then spread across LB agar plates to form a bacterial lawn. Plates were then incubated overnight, at 37 °C.^39^ The inoculated bacteria grew on the agar to an extent to which the sample was concentrated enough to inhibit the growth. In the both liquid method using the MTT assay ((3-(4,5-dimethylthiazol-2-yl)-2,5-diphenyl tetrazolium bromide), the bacteria (*E. coli* and *S. aureus*) were cultured in a 50 mL tube using 10 mL of LB with 10 mM MOF. The initial optical density (OD) was 0.1, and the cultures were incubated at 37 °C for 16 h. The negative control was LB with 10 mM MOF, and the positive control was bacteria in LB. Then, 100 μL of each sample was incubated in a microtiter plate, and 0.1 mg/mL MTT solution was added to each well and incubated at 37 °C for an additional hour. Finally, the culture with MTT was centrifuged to pellet the dark-blue crystals; then, the supernatant was removed, and the crystals were resuspended in dimethyl sulfoxide. The rate of bacteria viability was determined by measuring the absorbance at 570 nm. The experiments were repeated twice, and data were presented as ± SD.^40^ In the viability assay, two parameters were employed: the MTT assay and the analysis of the final OD after 16 h of incubation. This experiment utilized 4.6 mg L^–1^ EE2—*T*_f_ cycle 2; 4.7 mg L^–1^ EE2—*T*_f_ cycle 3; 4.9 mg L^–1^ EE2—*T*_f_ cycle 4; 5.6 mg L^–1^ EE2—*T*_f_ cycle 5; 6.0 mg L^–1^ EE2—*T*_f_ cycle 6; 7.5 mg L^–1^ E1—*t*_initial_; 1.1 mg L^–1^ E1—*T*_final_; 7.5 mg L^–1^ EE2—*t*_initial_; 4.1 mg L^–1^ EE2—*t*_intermediate_; and 3.4 mg L^–1^ EE2—*t*_final_ mixture in an LB medium, starting with an initial OD of 0.1 for each culture. The negative control consisted of an LB medium with each solution, excluding bacteria, while the positive control included bacteria in the LB medium.

### 2.5 Analytical Methods

High-performance liquid chromatography (HPLC) using a laser-induced fluorescence detector was the technique chosen to separate and evaluate the E1 and EE2 standards and their by-products. The HPLC method was developed and validated, as demonstrated by Oliveira and coauthors (2020).^9^

## 3 Results and Discussions

### 3.1 Morphological, Structural, and Spectroscopical Characterization of UiO-66

**Figure 2:**
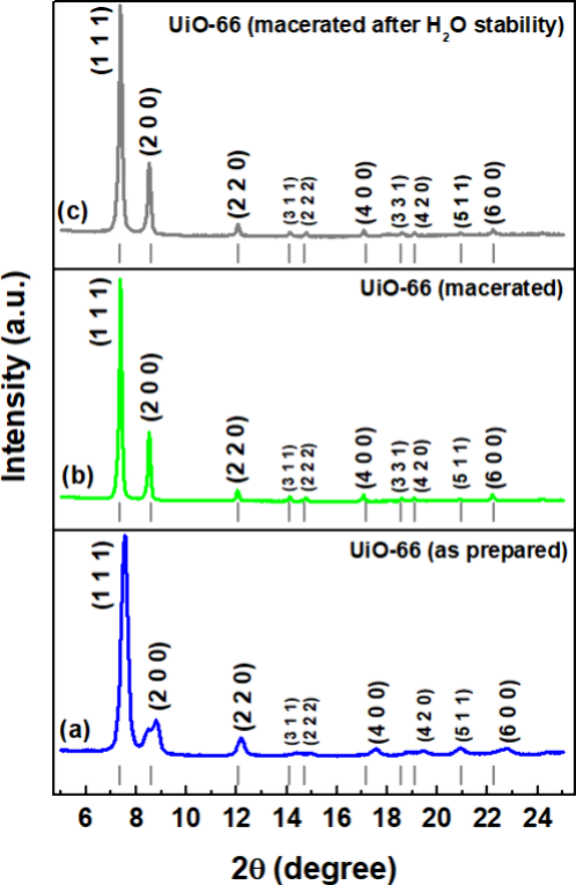
XRD patterns of (a) as-prepared UiO-66, (b) macerated UiO-66, and (c) macerated UiO-66 followed by a stability experiment in H_2_O for 24 h.

The properties of MOFs as well as water stability were evaluated using different analytical techniques such as X-ray diffraction (XRD) patterns, scanning electron microscopy (SEM), the BET method, and surface area. Figure 2 shows the XRD profile after the synthesis steps for UiO-66 production. The diffractogram presents an intense main peak at 2θ = 7.34°, which indicates the high crystallinity of the metallic complex, in addition to the other peaks at 8.6°, 12.06°, 17.16°, 19.11°, and 22.25°, which correspond, respectively, to the diffraction planes (111), (200), (220), (311), (222), (400), (331), (420), (511), and (600), confirming the formation of Zr-MOF UiO-66 associated with the group NH_2_.^41^ It is indexed with standard COD Card no. 1562103 (cubic, space group *Fm*3̅*m*, *a* = 30.203 Å), referring to UiO-66 (Zr).^42^ Wide peaks are observed right after synthesizing the metallic organic material, mainly at 2θ = 8.5°, indicating large crystallite sizes (Figure 2a). The UiO-66 underwent a mechanical process (maceration) to reduce the amount of clustered crystallites. After the process, a new XRD measurement was conducted to confirm its mechanical stability and crystallite size. As observed in Figure 2b, the peaks showed no shifts relative to the Zr-MOF UiO-66 after maceration. Furthermore, no additional peaks were observed upon comparison of the XRD diffractograms, indicating that the material remained stable even after being subjected to mechanical pressure. However, on comparison of the width of the diffraction peaks of the materials before and after maceration, a narrowing of the width and an increase in the intensity of the peaks can be noted when a decrease in the size of the nanoparticles occurs. The MOF particle size before and after the maceration process was calculated by the Debye–Scherer equation:
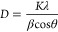
1where *K* is the Scherrer constant, λ is the length of the X-ray beam (1.54184 Å), β is the full width at half-maximum (fwhm) of the peak, and θ is the Bragg angle.^43^ The calculated crystallite size was ∼138.52 nm for the as-prepared UiO-66 nanoparticles and ∼32.47 nm for the material after the maceration process. The aqueous stability of the UiO-66 MOF was also investigated. After immersing UiO-66 in water for 24 h, the X-ray powder diffraction pattern of UiO-66 hardly changed, indicating that UiO-66 can remain stable in this medium (Figure 2c).

**Figure 3:**
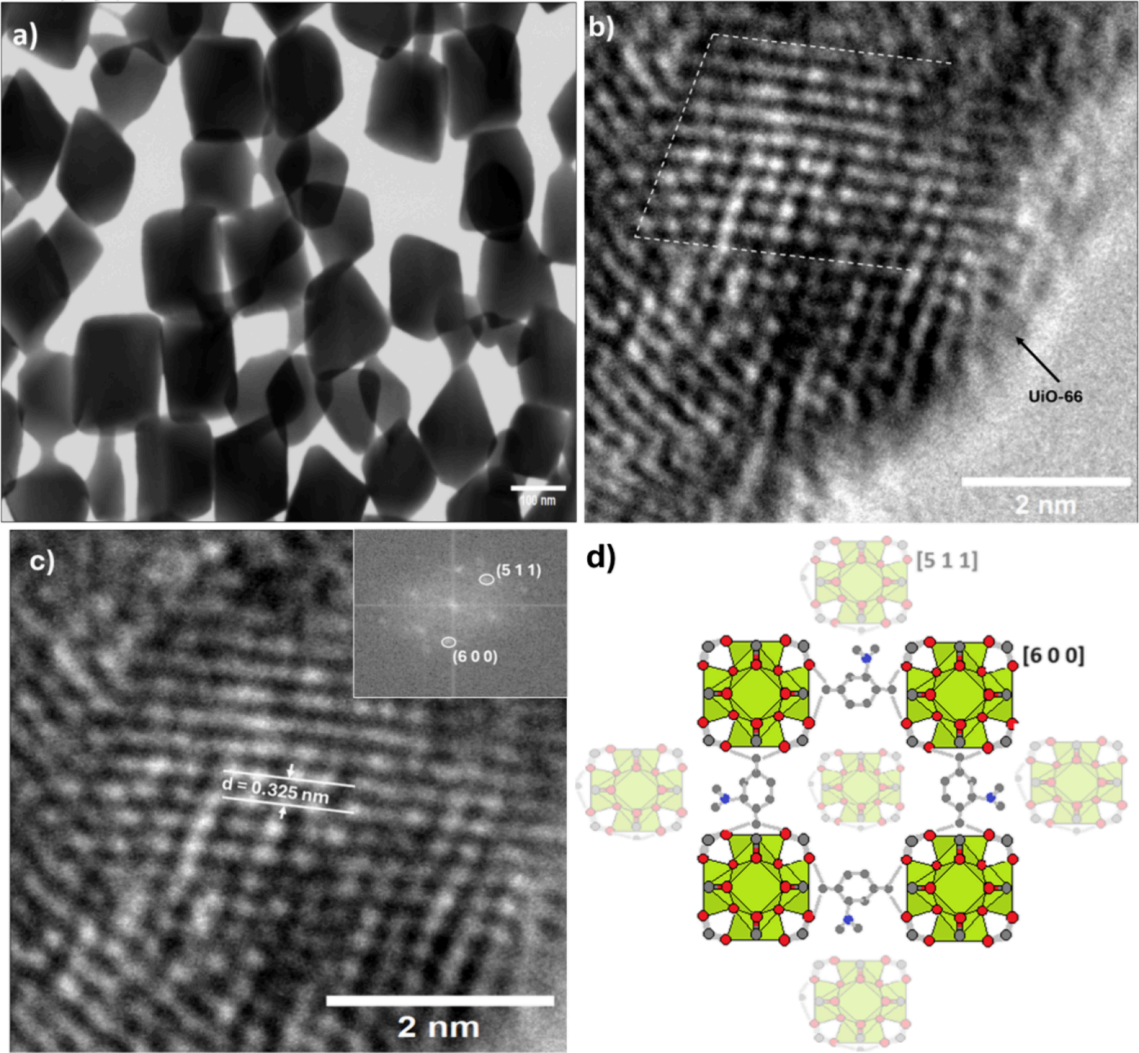
UiO-66-nanoparticles: (a) BF-STEM of uniform distribution particle size around ∼163.61 nm. (b, c) High-resolution TEM recorded at room temperature. The inset shows an FFT pattern of the HRTEM image. (d) Representative UiO-66 MOF structure (zirconium–oxygen cluster coordinated by terephthalic acid molecules) in the lattices of (6 0 0) and (5 1 1).

To confirm the synthesis and structure of UiO-66 MOF, STEM and HRTEM characterizations were carried out, as shown in Figure 3. The scanning TEM image in the bright-field mode (Figure 3a) showed distinctly the octahedral morphology of UiO-66 as-prepared nanoparticles, in which the nanoparticles presented a homogeneous size of ∼163.61 nm. In Figure 3b–d, the structure was directly related to the TEM images. The interplanar spacing shown in Figure 3b corresponds to the (6 0 0) UiO-66 MOF cubic structure (0.325 nm), which was estimated by the fast-Fourier transform (FFT) pattern after the deconvolution of signs. The framework illustrated in Figure 3d is similar to the HRTEM image in the (6 0 0) zone axis. Based on the symmetry of the related space group, a crystallographic reconstruction typical of real space was carried out for UiO-66 via HRTEM imaging. From the amplitudes and phases of the HRTEM reflections obtained along (6 0 0), it was possible to obtain the (5 1 1) plane via symmetry operations in the *Fm*3̅*m* space group.

Next, the N_2_ adsorption/desorption isotherms on the synthesized MOF were investigated to identify the porous properties, surface area, and pore volume (Figure S1). The UiO-66 MOF before and after maceration showed similar isotherm shapes according to Figure S1a,c, respectively. In addition, a steep increase in N_2_ uptake at low relative pressure (*P*/*P*_s_ ≤ 0.01) indicates that all of the samples are highly nanoporous. No hysteresis is observed for the UiO-66 MOF before and after the maceration process (Figure S1a,c). The absence of a small hysteresis at 0.8 and 0.4 relative pressure (Figure S1c) means that no unreacted reactants are deposited in the pores and the variation of pore size in the MOF is negligible (Figure S1b,d), respectively.^44^ The temperature of heat treatment to avoid the presence of interferents inside the pores was chosen based on a previous study carried out at 80 °C in the UiO-66 after maceration. This study observed a small hysteresis at 0.8 and 0.4 relative pressures, as shown in Figure S1e,f.

Furthermore, the N_2_ adsorption isotherms at 120 °C also suggest that the as-prepared UiO-66 has a greater pore volume and a smaller surface area (0.82 and 1004.67 m^2^/g, respectively) than that after maceration (0.61 and 1204.89 m^2^/g, respectively). Such measurements were obtained using the BET method. The maceration process reduced the pore volume of the UiO-66 particles by 1.3 times, accompanied by a significant increase in the surface area.

To confirm the UiO-66 presence, FTIR analysis of the UiO-66 powder synthesized after heat treatment was also performed, as shown in Figure S2. The presence of bands corresponding to O–H and N–H vibrations originated by the organic ligand at 3391–2999 cm^–1^ is observed. The characteristic bands of UiO-66 are also identified, relating to the asymmetric and symmetric stretching of the C=O bonds at 1700 cm^–1^ and COO^–^ at 1600–1500 cm^–1^. The band at 800–600 cm^–1^ represents the Zr–O bond,^45,46^ indicating the occurrence of the coordination reaction between the −COOH of the ligand and the Zr^4+^ of the metal cluster. Furthermore, the peaks at 1381–1251 cm^–1^ correspond to the C–N bonds of aromatic amines, demonstrating the presence of the functionalizer in the structures of the nanoporous cavities.^45^ Thus, FTIR spectroscopy also confirmed the synthesis of the UiO-66 MOF associated with the NH_2_ group, which corroborated with XRD analysis.

**Figure 4:**
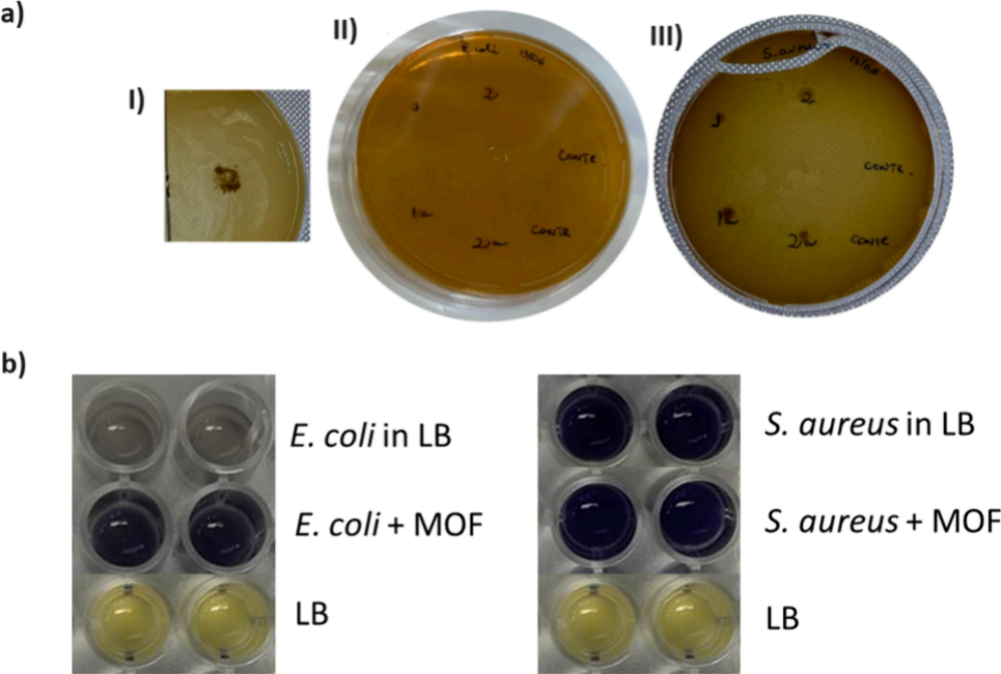
(a) Agar disc diffusion results for the antibacterial activity study of (I) MOF as a substrate using (II) *E. coli* and (III) *S. aureus*. (b) Broth liquid method using the MTT assay for MOF antibacterial analysis for *E. coli* and *S. aureus*. Photograph of the biotoxic experiment of UiO-66 on bacteria. May 2024. Taken by the Authors.

Considering the application of the UiO-66 in the system of supply water treatment, the antibacterial activity of UiO-66 MOF against *E. coli* and *S. aureus* bacteria cells was investigated using two approaches, “agar disk diffusion” and “broth liquid method using MTT assay.” In the agar disk diffusion assay, the antibacterial activity of UiO-66 was not detected, as confirmed by the absence of any zone of inhibition (Figure 4a). A similar result was obtained in the MTT assay. This consists of the evaluation of the mitochondrial metabolism of cells by observing the reduction of 3-(4,5-dimethylthiazol-2)-2,5-diphenyltetrazolium bromide, also known as tetrazolium bromide, thiazolyl blue, or MTT, producing formazan. MTT has a yellow color, while formazan, resulting from its reduction, has a purple color. The change in the color of the reaction can be evaluated through a spectrophotometer (Figure 4b).^47^

### 3.2 Characterization of Self-Organized TiO_2_ Nanotubular Arrays Modified with UiO-66 by Spray-Coating and Drop-Casting Methods

**Figure 5:**
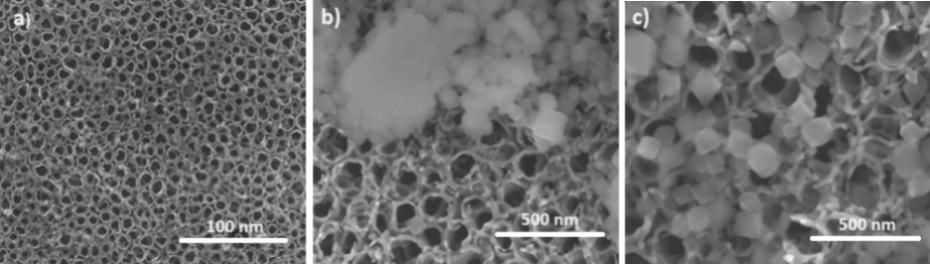
SEM images of morphology: (a) Nt/TiO_2_ without modification and with UiO-66 nanoparticles deposited by (b) drop-casting and (c) spray-coating methods.

The electron microscopy images (Figure 5) show the morphology of the oxide film grown on the pure Ti substrate under the anodization process, without and with modification of UiO-66 nanoparticles by drop-casting (Figure 5b) and spray-coating (Figure 5c). The drop-casting nanoparticle deposition process reveals significant agglomeration of the MOF structures, attributed to nucleation between the UiO-66 networks within the deposition solution.^48^ On the other hand, employing the spray-coating method for modification enhanced the dispersion of UiO-66 MOF nanoparticles onto the nanotubes, mitigating the particle agglomeration phenomenon. A similar approach was adopted by Ahmadi et al., where they achieved uniform deposition through solution spraying with nanoparticles suspended in a solution.^49^ For both deposition methods, MOF nanoparticles had an average size of ∼41.7 nm, according to the size distribution shown in Figure S3a, calculated from the ratio between the standard deviation of the nanoparticle size distribution and the average size of approximately 200 nanoparticles. In addition, the SEM image shows that the synthesized nanoparticles presented an octahedral morphology with well-defined facets (Figure S3b). The octahedral morphology of the MOF is one of the characteristics that confer water stability and high selectivity in the adsorption of molecules.^47^ This morphology depends on the physicochemical parameters of synthesis, as well as the choice of the metallic cluster and organic ligand.^50^ With a particle size distribution of around 40 nm, UiO-66 nanoparticles could penetrate the cavities of the nanotubes, which possess an internal diameter slightly larger than the average size of the octahedral particle. This uniform distribution was possible due to the maceration process of the as-prepared UiO-66 and the complete homogenization of the solution used in the deposition process.^49^

**Figure 6:**
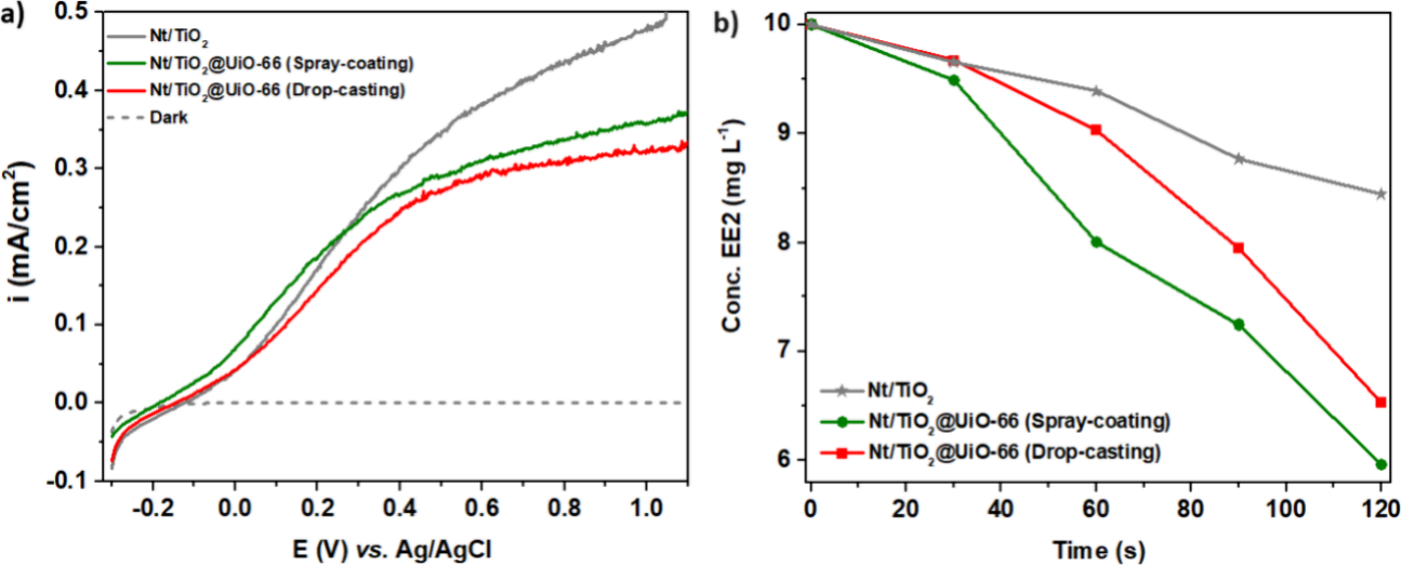
Comparison of Nt/TiO_2_@UiO-66 decorated using spray-coating (green line) and drop-casting (red line): (a) linear sweep voltammetry performed under UV–vis irradiation in 0.2 M Na_2_SO_4_ (pH 5.7) with a scan rate of 10 mV/s; and (b) photocatalytic degradation under UV–vis irradiation for EE2 during 120 s using a solution of 10 mg L^–1^ EE2 diluted in distilled water (pH 7.0).

In Figure 6a, photoactivity studies of Nt/TiO_2_@UiO-66 are presented, comparing the deposition methods of UiO-66 nanoparticles: drop-casting (red line) and spray-coating (green line). Additionally, the current density values of the unmodified Nt/TiO_2_ electrode are represented (gray line). For both methods, the current density is reduced with the deposition of UiO-66 nanoparticles on the nanotube layer. This occurs because this MOF is not photoactive, and its presence on the surface of the nanotubes reduces the available area of light incidence and consequently reduces the photoactivity of the electrode. Given the similar reduction in current density observed with both methods of UiO-66 nanoparticle deposition, a more insightful comparison was conducted through a degradation study of EE2 (10 mg L^–1^) under UV–vis radiation for 120 s. Degradation percentages of 16, 35, and 41% were achieved for Nt/TiO_2_, Nt/TiO_2_@UiO-66 (drop-casting), and Nt/TiO_2_@UiO-66 (spray-coating), respectively, as shown in Figure 6b. These results suggest that the spray-coating method performs better than the drop-casting method, probably due to its ability to achieve a more uniform dispersion of nanoparticles. Although these nanoparticles enhance shading of the nanotubes under irradiation, the proximity of pollutants to the hydroxyl radicals generated on the photocatalyst compensates for the photoactivity reduction.

### 3.3 Morphological and Compositional Characterizations of the Self-Organized Nanotubular Oxide Layer on Ti–W Alloys Modified with UiO-66

To evaluate the morphology and distribution of UiO-66 nanoparticles on a self-organized nanotubular oxide layer on the Ti-xW alloy, SEM and EDS analyses were carried out, and the results are shown in Figure S4. A uniform and homogeneous distribution of the UiO-66 nanoparticles on the nanotubes is observed (Figure S4a). Small particles can be seen within the inner diameter of the nanotubes, forming a coupling between the MOF and the oxide layer that extends along the material’s surface. The EDX analysis identified the presence of Ti, W, O, Zr, C, and O elementals in the composition; the homogeneous distribution of Zr on the oxide layer (Figure S3b), together with the presence of Ti, W, and O (Figure S4b,c), which are related to the oxide layer, can be observed. No impurities were detected.

**Figure 7:**
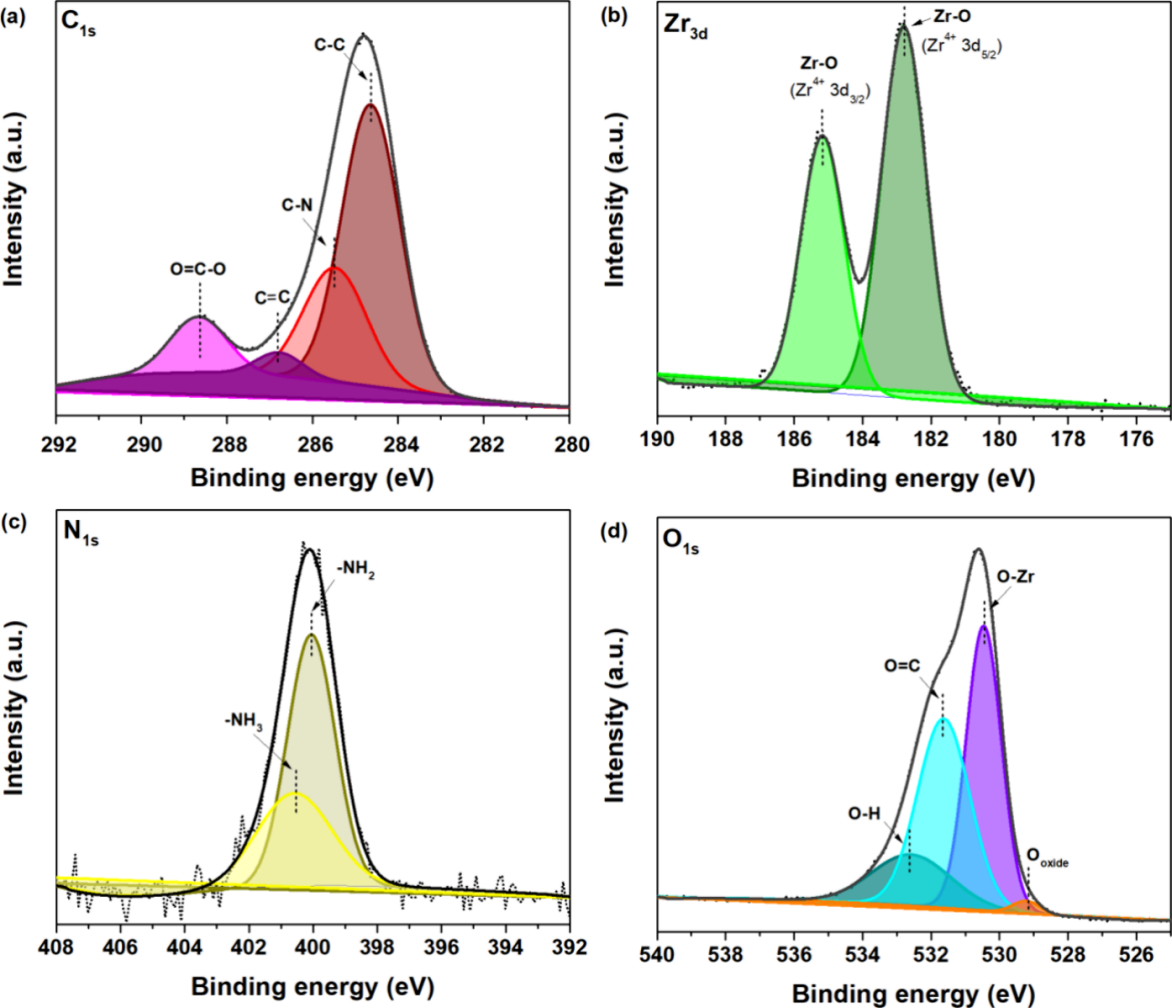
High-resolution deconvolution of (a) C 1s, (b) Zr 3d, (c) N 1s, and (d) O 1s spectra from the sample of Nt/Ti-5.0W@UiO-66 before photoelectrocatalysis application.

XPS analyses were conducted on Nt/Ti-*x*W@UiO-66 and are presented in Figure 7. The spectra of Nt/Ti-0.5W and Nt/Ti-5.0W electrodes without deposition of UiO-66 nanoparticles were presented and discussed by Oliveira et al. and de Almeida et al., where the presence of TiO_2_ and stoichiometric and nonstoichiometric W oxides was observed in the oxide composition.^6,9^ The high-resolution spectra in the C 1s, Zr 3d, N 1s, and O 1s regions revealed the presence of peaks, confirming the oxidative species of C, Zr, N, and O in the composition of the sample synthesized on the nanotubes grown on Ti-5.0W alloy. Figure 7a shows the contributions of carbon species associated with the bonding units. Deconvolution revealed the prominent presence of C–C (284.6 eV), C–N (285.4 eV), C=C (286.8 eV), and O=C–O (288.6 eV), according to the oxidative species that make up the UiO-66. Regarding the spectrum of Zr 3d (Figure 7b), the peaks at 182.7 and 185.1 eV can be attributed to Zr^4+^ 3d^5/2^ and 3d^3/2^,^51^ respectively, suggesting the proper formation of Zr–O bonds in the metal cluster. Figure 7c presents the N 1s spectrum, showing the peaks related to the contribution of −NH_2_ bound to the phenyl ring of the ligand (−NH_2_, 400.0 eV) and the protonated form of the amidogen (−NH_3_^+^, 400.5 eV), resulting from atmospheric oxidation due to the interaction of ammonia with the hydroxyl radical (H_2_O–NH_3_^+^).^52^ The deconvolution of the O 1 s spectrum (Figure 7d) reveals the O bonds in the formation of the UiO-66 MOF, showing contributions through the peaks associated with C=O (531.8 eV) and Zr–O (530.3 eV).^51,53^ Additionally, the presence of the semiconductor is evident in the sample composition, as indicated by the peak observed in the O 1s spectrum, corresponding to oxygen linked to oxides (529.1 eV).^54^ Finally, the chemical composition of the surface in terms of the molar ratio of O/Zr and C/Zr (5.4 and 7.5, respectively) follows the stoichiometry corresponding to the C_46_H_34_N_2_O_32_Zr_6_ unit cell (i.e., 5.3 and 7.6, respectively). The experimental N/Zr ratio was slightly higher than the theoretical stoichiometry (3.5 and 3.0, respectively). This slight deviation may be attributed to bonds originating from unreacted precursors.

### 3.4 Photoelectrocatalytic Hormone Degradation

**Figure 8:**
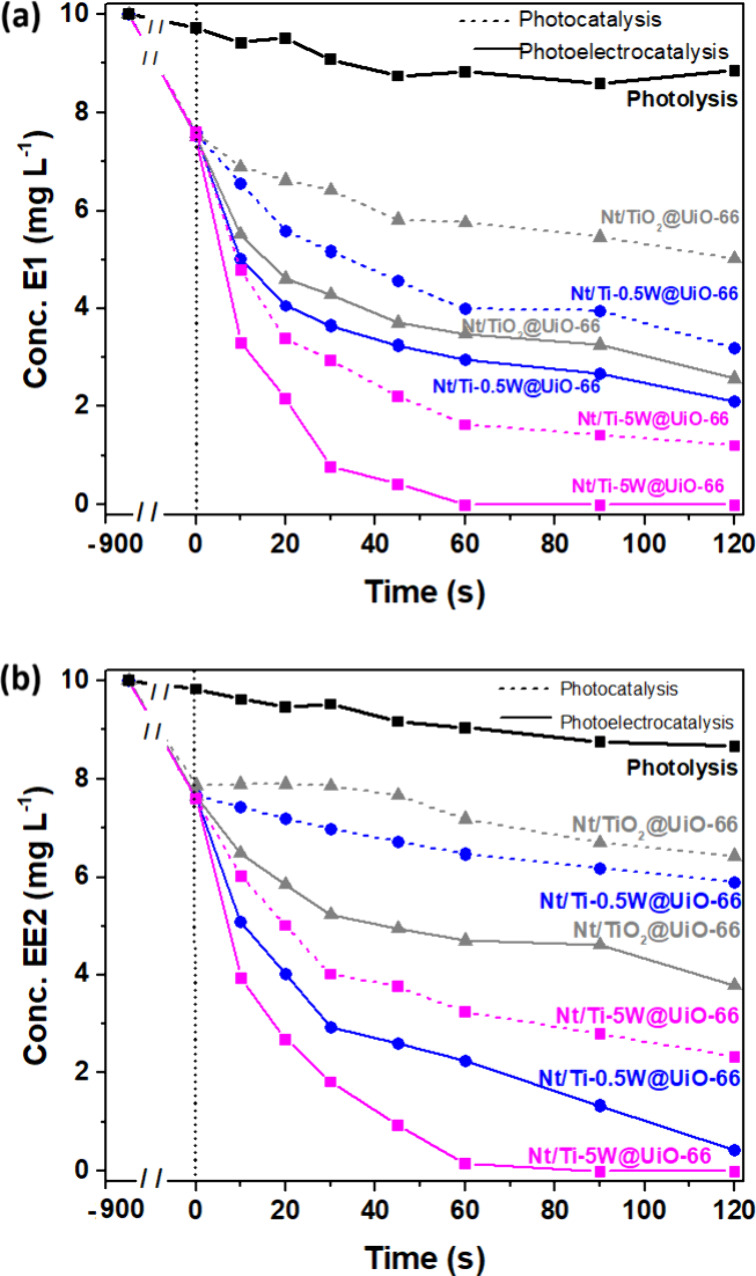
Degradation performance comparison of (a) E1 and (b) EE2 by photo(electro)catalysis under UV–vis irradiation using Nt/TiO_2_@UiO-66, Nt/Ti-0.5W@UiO-66, and Nt/Ti-5.0W@UiO-66 as the photoelectrode. Initial condition: 10 mg L^–1^ E1 or EE2 diluted in distilled water, pH 7.

Figure 8 compares the E1 and EE2 photocatalytic and photoelectrocatalytic degradation curves using the Nt/TiO_2_@UiO-66, Nt/Ti-0.5W@UiO-66, and Nt/Ti-5.0W@UiO-66 electrodes. Before the photodegradation system was irradiated, the adsorption equilibrium between the photocatalyst modified with UiO-66 and the hormone in the dark was conducted for 15 min. This time was established according to the adsorption profile shown in Figure S5 (SI). For all electrodes, regardless of the type of hormone, after 15 min of interaction, the concentration of the organic compound showed a nonsignificant variation. Figure 8a presents the results of the photocatalytic and photoelectrocatalytic removal of E1 in synthetic water and its comparison with the photolysis, control experiment employing only UV–vis irradiation (λ > 320 nm). Under only radiation, a degradation of the E1 molecule of less than 10% occurred after 60 s, reaching a removal of ∼15% after the total monitoring time (120 s). This percentage corroborates with the literature, which showed an insignificant contribution of photolysis in removing E1 under UV–vis radiation.^11,55^ E1 is one of the compounds most susceptible to UV radiation among estrogens due to its high molar absorption coefficient (402.4 M^–1^ cm^–1^) and quantum yield of 0.065 mol E^–1^ at λ = 253.7 nm.^10^ Therefore, instability of the E1 molecule under UV–vis radiation is expected.

The association of radiation and the semiconductor, called the photocatalytic process, demonstrated the inhibition of estrogenic activity in the following decreasing order with the type of electrode used: Nt/Ti-5.0W@UiO-66, Nt/Ti-0.5W@UiO-66, and Nt/TiO_2_@UiO-66, achieving removal rates of 83.6, 60.4, and 42.0%, respectively, within 60 s. However, when voltage is applied to the system to favor the flow of negative charges, the recombination of photogenerated charges is avoided, and the inhibition of estrogenic activity increases, reaching a total removal after 60 s of treatment using the Nt/Ti-5.0W@UiO-66 electrode. A similar behavior is observed in the EE2 removal profiles in synthetic water using Nt/TiO_2_@UiO-66, Nt/Ti-0.5W@UiO-66, and Nt/Ti-5.0W@UiO-66 photoanodes, as shown in Figure 8b. The complete inactivation of EE2’s estrogenic activity occurred within 60 s of treatment through the photoelectrocatalytic process employing the Nt/Ti-5.0W@UiO-66 electrode. Notably, the Nt/TiO_2_@UiO-66 and Nt/Ti-0.5W@UiO-66 electrodes demonstrated reductions of 53.1 and 77.3%, respectively, in EE2 hormone levels under the same process. These results were superior to those obtained via photocatalysis, in which the maximum removal level was around 67.7% in the presence of Nt/Ti-5.0W@UiO-66. When comparing these results with the performance of Nt/Ti-*x*W photoanodes without modification by UiO-66 MOF nanoparticles, as investigated by de Almeida et al. and Oliveira et al., it becomes apparent that the incorporation of MOF onto the nanotubes leads to a reduction in the removal efficiency of E1 and EE2 in synthetic water.^6,9^ Specifically, this reduction is noted to be 3.3, 2.3, and 2.1 times greater compared to nanotubular films of Nt/TiO_2_, Nt/Ti-0.5W, and Nt/Ti-5.0W, respectively, after a 60 s photoelectrocatalysis process. The addition of UiO-66 MOF led to an average enhancement of 40% in the removal process, likely due to the greater sorption of the hormone by the MOF, which brought it closer to the photogenerated oxidative agents at the metal/solution interface. Consequently, this sorption process contributed to the degradation process by the Nt/TiO_2_ and Nt/Ti-*x*W oxide layers occurring quickly. In both photocatalytic and photoelectrocatalytic treatments, the hierarchy of electrode efficiency persisted, regardless of the presence or absence of UiO-66 MOF. The only noticeable change was the increase in degradation efficiency over treatment time by modifying nanotubular oxides.

### 3.5 Hormone Degradation in a Real Water Matrix

**Figure 9:**
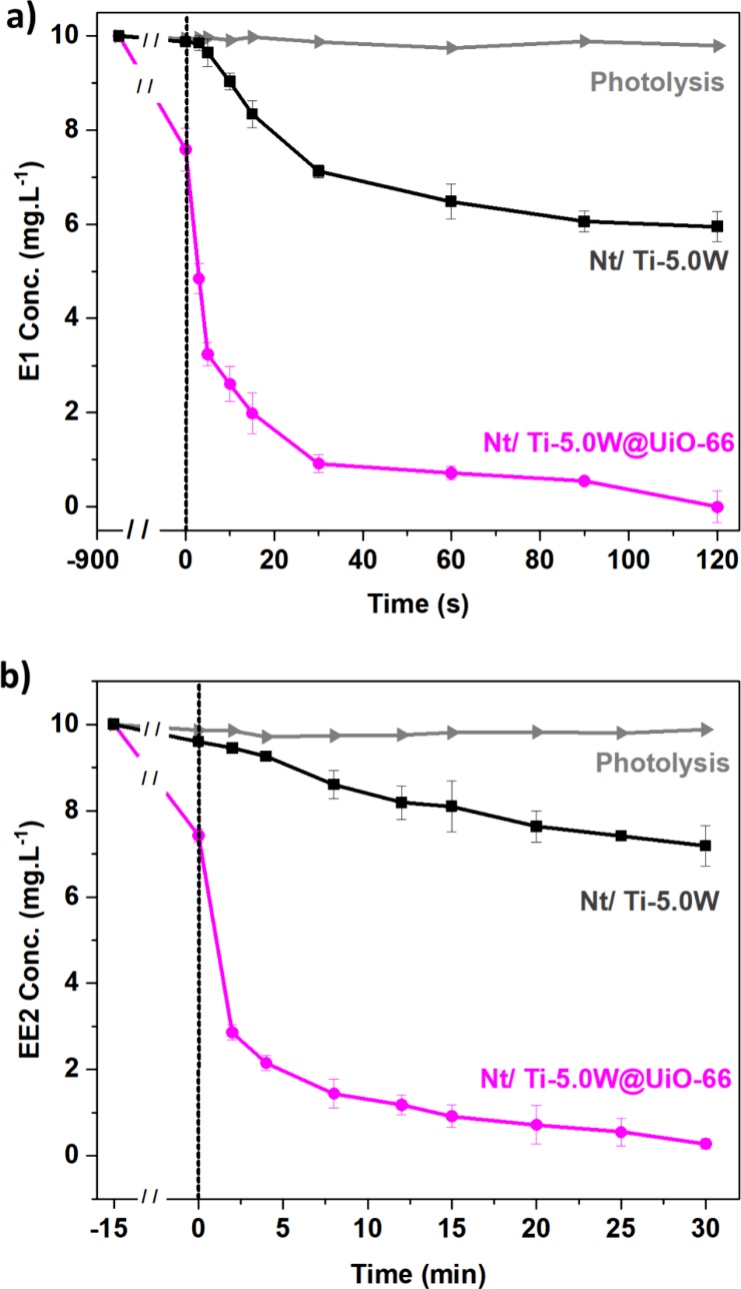
Photoelectrocatalytic degradation of 10 mg L^–1^ (a) E1 or (b) EE2 diluted in a real water matrix under UV–vis irradiation using Nt/Ti-5.0W and Nt/Ti-5.0W@UiO-66, pH 7.5.

The electrode with the best performance in removing E1 and EE2 in synthetic water, with and without modification with UiO-66 (Nt/Ti-5.0W@UiO-66 and Nt/Ti-5.0W), was evaluated for the photoelectrocatalytic removal of these hormones in a real water supply matrix under UV–vis radiation. Figure 9 shows the concentration profiles of E1 (Figure 8a) and EE2 (Figure 8b) as a function of reaction time in the presence of photocatalysts Nt/Ti-5.0W and Nt/Ti-5.0W@UiO-66. In the presence of MOF, there is a significant adsorption process before starting the photoelectrocatalytic treatment, reducing the concentration of hormones in solution by around 27%. After the incidence of radiation and potential application, an exponential decline is observed for both hormones. However, due to the complexity of the real water matrix, the time required to remove the hormones is greater than that obtained in synthetic water (Figure 9). For E1, after 60 s, only 80% of the hormone is degraded, reaching complete degradation after 120 s, twice the time required for synthetic water. In the real water matrix, the removal time for EE2 was significantly prolonged, with ∼90% reduction in EE2 concentration observed only after 15 min of reaction. This prolonged duration may be attributed to the competition of the target pollutant with other organic compounds within the water supply, which exhibited a total organic carbon load of 40 mg L^–1^. The presence of additional compounds can reduce the degradation efficiency of hormones due to parallel reactions with hydroxyl radicals. These compounds may include organic matter or inorganic anions such as chloride, bicarbonate, and sulfate.^56^ Moreover, the concomitant adsorption of certain inorganic ions or other dissolved organic compounds in the water matrix can also compete with the targeted pollutant.^10^

The hormone inactivation reactions (E1 and EE2) were more accelerated in the presence of the adsorbent material, demonstrating a collaborative effect between the adsorbent and the photocatalyst. This collaboration arises from the rapid consumption of oxidizing agents generated in the photocatalyst by pollutants and accumulated by-products closer to the electrode. This phenomenon minimizes mass transport via diffusion of organic species. Additionally, this assembled architecture consists of overlapping materials that are held together by noncovalent interactions,^57^ involving only electromagnetic interference, without any bond formation between the materials, sharing electrons, or electron transfer. Due to the nonphotoactivity response of the UiO-66, the electron transfer interaction or charge trapping between the materials is not feasible within the species,^58^ even after the decoration of Nt/Ti-5.0W with this MOF.

The Langmuir–Hinshelwood mechanism was used to characterize the heterogeneous photoelectrocatalytic reactions that occur on the surface of the Nt/Ti-5.0W@UiO-66 electrode during the degradation of E1 and EE2 present in the water supply. Four steps were considered: (1) adsorption of E1/EE2 molecules by the MOF (UiO-66), (2) reactions of the pollutant molecules adsorbed on the Nt/Ti-5.0W and UiO-66 MOF, (3) desorption/readsorption of by-products by the UiO-66, and (4) degradation reactions of by-products on the surface of the photocatalyst. Considering that the degradation reactions of organic compounds initiate only after absorption and desorption equilibrium, the Langmuir–Hinshelwood model in equilibrium can be expressed by the following equation (eq 2):
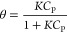
2where θ is a fraction of the coverage site, *K* is the adsorption–desorption equilibrium constant, and *C*_P_ is the organic pollutant concentration. Furthermore, the E1 and EE2 removal data obtained under ideal photoelectrocatalytic conditions in supply water were adjusted to the pseudo-first-order kinetic model according to eq 3, where *K*_1_ is considered the reaction constant between the variation in the organic pollutant concentration in relation to time.
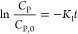
3

The results presented in Table 1 demonstrate that the degradation kinetics of photoelectrocatalysis are consistent with those of the proposed model. The correlation coefficient exceeds 0.99, and the χ^2^ values are acceptable (3 degrees of freedom, α = 0.05, at a critical value of 7.815), indicating a good fit of the pseudo-first-order kinetic model to both experimental and calculated data. Notably, the degradation rate constants (*K*_1_) show a significantly higher value for E1 when using the Nt/Ti-5.0W@UiO-66 electrode (*k* = 0.168 min^–1^), which is 1.78 times faster compared to the Nt/Ti-5.0W photocatalyst without the adsorbent material (*k* = 0.094 min^–1^). A similar behavior was observed for the removal of EE2. However, the degradation rate obtained for the removal of EE2 in the water supply using the photocatalyst coupled to the UiO-66 MOF (*k* = 0.310 min^–1^) was significantly higher, around 18.21 times greater than that of the unmodified photocatalyst (*k* = 0. 017 min^–1^).

**Table 1:** Kinetic Parameters of the Photoelectrocatalytic Degradation of E1 and EE2 under UV–Vis Irradiation

hormone	electrode	kinetic rate constant	*R*^2^	time_1/2_
E1	Nt/Ti-5.0W	0.094 s^–1^	0.993	73.780 (s)
	Nt/Ti-5.0W@UiO-66	0.168 s^–1^	0.997	4.105 (s)
EE2	Nt/Ti-5.0W	0.017 min^–1^	0.999	38.655 (min)
	Nt/Ti-5.0W@UiO-66	0.310 min^–1^	0.992	2.235 (min)

Compared to other results in the literature, the Nt/Ti-5.0W@UiO-66 heterojunction exhibited a considerably higher degradation rate of organic compounds in comparison to that of Xiaobo et al. This work used a suspension containing 0.002% TiO_2_ combined with HKUSRT-1 MOF (Cu-MOF) under UV radiation to remove methylene blue textile dye from real wastewater, reaching a kinetic rate of 0.032 min^–1^. This performance is approximately 10 times lower than the degradation rates achieved by the Nt/Ti-5.0W@UiO-66 electrode during EE2 removal from the water supply.

When comparing the performance of the Nt/Ti-5.0W@UiO-66 photocatalyst for removing hormones E1 and EE2 in synthetic water, the electrode demonstrated similar times for inactivating both hormones, achieving total removal of both compounds in less than 2 min of reaction. However, when the method was applied to a real water matrix, the removal times differed significantly: E1 required 2 min for complete removal, whereas EE2 required 30 min. This discrepancy may be attributed to the size difference between the molecules and their by-products. E1 has a molecular size of 0.89 nm, while EE2 equals 1.46 nm, making it approximately 1.64 times larger than E1. During the degradation process, E1 breaks down into smaller molecular fragments, whereas EE2 degradation involves the formation of denser intermediates. Additionally, the degradation pathway of EE2 includes more steps than E1, leading to the formation of lighter by-products as described in Oliveira.^9^

According to Huang et al., porous structures can accommodate more molecules depending on the size of their cavities, facilitating the adsorption of pollutants from the solution.^59^ The UiO-66 cavities can accommodate more E1 molecules and their by-products, accelerating their degradation. On the other hand, EE2 interacts less favorably with UiO-66 due to its larger size and the formation of denser by-products, which can delay diffusion. In synthetic water, where no competing organic compounds exist, the size and density differences between E1 and EE2 by-products do not significantly impact their degradation rates. However, in a real water matrix, smaller organic molecules present in the environment can more readily occupy the UiO-66 cavities, reducing the availability of EE2. This competition decreases the degradation rate of EE2 compared to E1. Additionally, the density of EE2 by-products interferes with their diffusion, making it difficult to capture and degrade new EE2 molecules.

### 3.6 Recyclability and Stability Test

**Figure 10:**
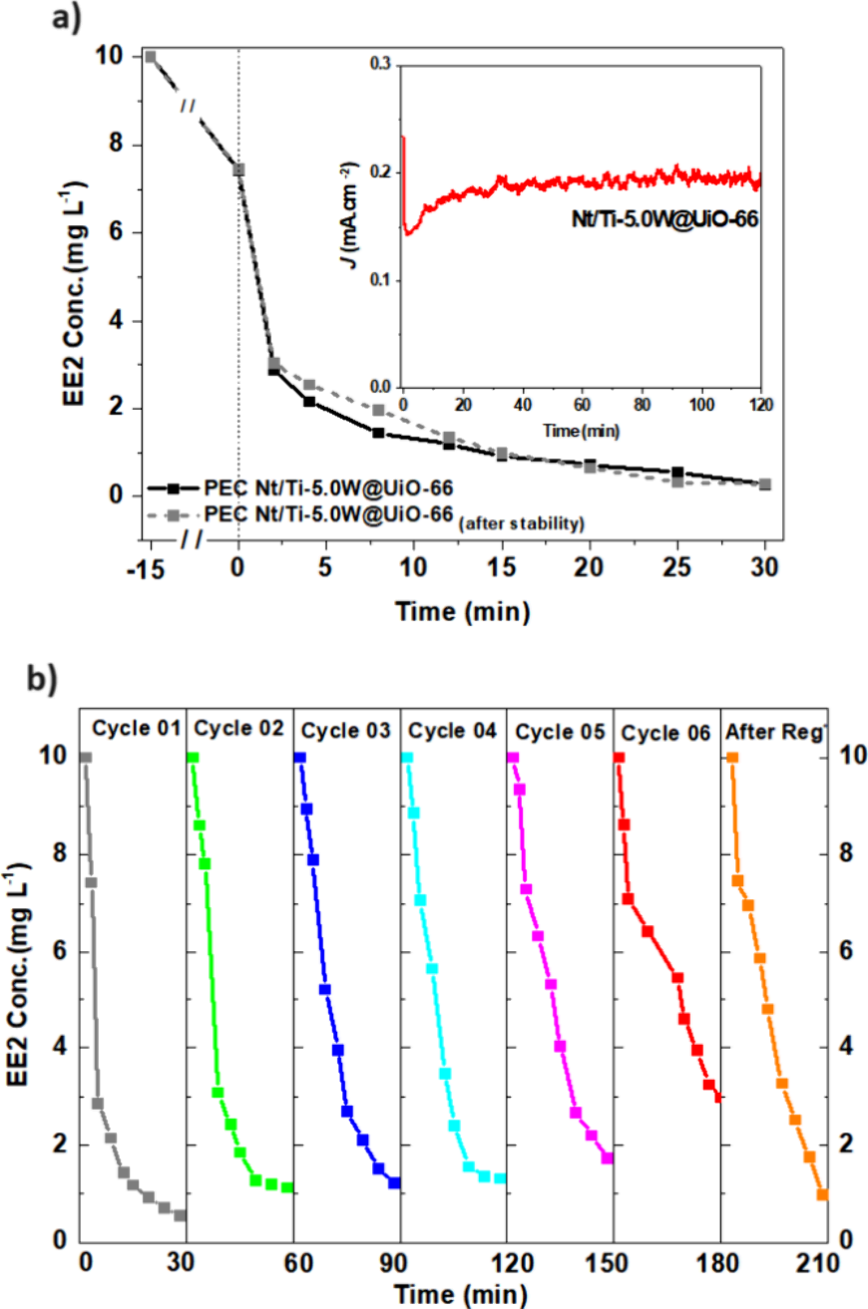
Stability of the Nt/Ti-5.0W@UiO-66 electrode for photoelectrocatalytic degradation of EE2 in matrix water under UV–vis irradiation at 1.3 V: (a) comparison between electrode performance before and after (inset) the chronoamperometric experiment under light irradiation and constant potential during 120 min; and (b) recycling tests of degradation followed by regeneration of the electrode after six cycles.

Reusing the photocatalyst is an important parameter because it reduces the degradation process’s costs and reveals promising materials for photocatalytic processes. Then, Nt/Ti-5.0W modified with UiO-66 nanoparticles was submitted to different stability tests, and the results are presented in Figure 10. Pollutant degradation experiments carried out before and after a prolonged stability test using the chronoamperometric method (inset: Figure 10a) under electrochemical oxidation conditions demonstrated similar degradation profiles with an EE2 removal efficiency of around 90% after 20 min of treatment (Figure 10a). Moreover, the stability test showed consistent current density over time, indicating minimal susceptibility to photochemical corrosion. This resistance can be attributed to the strong covalent bonds between the elements in the Ti–O–W oxides and UiO-66 MOF that constitute the photocatalyst. These bonds ensure the chemical integrity of the material, while the morphology of UiO-66 enhances its mechanical stability.^31^

Regarding the recycle tests of Nt/Ti-5.0W@UiO-66, six cycles of photoelectrocatalytic degradation of EE2 in a real matrix under UV–vis radiation at 1.3 V were conducted, and the data are compared in Figure 10b. While the degradation rates in cycles 1–4 show minimal changes, there is a noticeable gradual decline in the performance of the Nt/Ti-5.0W@UiO-66 electrode after successive 30 min cycles. The efficiency of hormone degradation decreases significantly after 6 cycles, reducing from approximately 95% to about 70% degradation within 30 min. This decline suggests that over successive treatments, some EE2 molecules and their intermediates may become adsorbed or trapped within the pores and cavities of UiO-66 MOF, thereby reducing the photocatalyst’s activity. Prolonged use of the electrode for 120 min contributed to the oxidation of all organic molecules adhered to the active sites of the composite material (Nt/Ti-5.0W@UiO-66), promoting the regeneration of the photoelectrode. This phenomenon was validated by achieving performance similar to that of the initial cycle immediately after the regeneration test of the Nt/Ti-5.0W@UiO-66 electrode. Consequently, the electrode demonstrated stability and proved to be a promising material for applications in photocatalytic processes for treating water supplies with low concentrations of pollutants.

To investigate the structural stability and durability of the synthesized MOF after cycle tests, XPS (Figure S6) and XRD (Figure S7) analyses were carried out for the electrode. The comparison between the XRD profile of Nt/Ti-5.0W@UiO-66 as-prepared and after six cycles did not show structural changes, indicating high stability and durability. For the recuperation process, the electrode was submitted to a chronoamperometry experiment for 2 h, in which all organic compounds were effectively removed from its matrix. However, this treatment resulted in a shift in the diffraction peaks related to UiO-66 MOF, as observed in Figure S7. However, the crystallinity was maintained.

Also, XPS analyses were carried out to check and compare with the X-ray diffractogram, according to Figure S6c–f (SI). Compared to XPS spectra carried out on the same samples, a reduction of C–N binding can be observed, which indicates the oxidation of the functionalized amine group during the photoelectrocatalytic removal of hormones. According to Kaur et al., the amine groups undergo oxidation and sacrifice their e–, preventing the oxidation of the ligands of the MOF structure and consequently preserving the MOF structural integrity.^60^ This behavior is clarified by the recycling tests that showed electrode efficiency reduction for each experiment. The functionalized amine group has an effect on accelerating the reactions in the photocatalytic process,^61^ and then, after all amine is sacrificed, the electrode performance decreases even after the pollutant depletion from the cavities and pores of the MOF, as observed in Figure 10b (curve after regeneration). The consumption of the entire amine group observed by XPS analysis is corroborated with the XRD data, which showed a shift in the MOF peaks after the electrode recovery process.

### 3.7 The Confinement Effect and Photodegradation Mechanism

**Figure 11:**
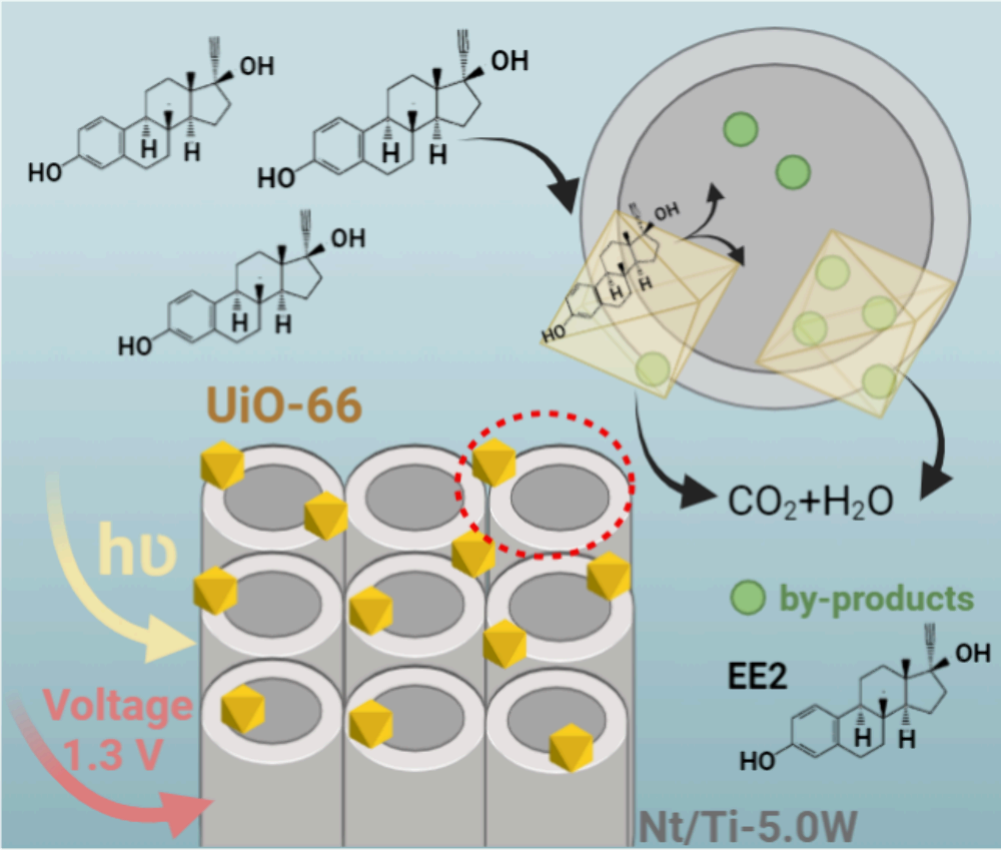
Mechanism of the photoelectrocatalysis reaction of the hormone EE2 using the Nt/Ti-5.0W electrode modified with a UiO-66 MOF.

Figure 11 proposes a possible mechanism for the interfacial behavior between the adsorbent and photocatalyst based on degradation tests (Figure 9) and XPS analysis (Figure S6a,b) conducted on the Nt/Ti-5.0W@UiO-66 electrode after photoelectrocatalytic treatment of real water supplies containing EE2. The slower degradation of EE2 compared to E1 enables a more careful assessment of the conditioning of organic compounds into the adsorbents.

In the presence of UiO-66 MOF, a pore channel of 2–4 nm uniformly distributed over the Nt/Ti-5.0W is produced, increasing the confinement effect of the pollutant and bringing it closer to the surface of the photocatalyst. The cooperative effect between Nt/Ti-5.0W and UiO-66 is evident when the photoelectrocatalytic degradation rates of E1 and EE2 with and without UiO-66 are compared (Table 1). It is observed that these materials function complementarily: UiO-66 adsorbs hormones from the solution via electrostatic attraction facilitated by NH_3_ groups within the UiO-66 MOF structure,^51^ while Ti–O–W nanotubes produce photogenerated OH^·^ radicals essential for hormone degradation. As a result of this collaboration, we observe a rapid initial reduction of the pollutant EE2 within the first 12 min of photoelectrocatalytic treatment (Figure 9b), followed by a slower degradation until complete removal. This deceleration is attributed to the formation of by-products that remain closely associated with the adsorbents, showing a preference for adsorption over EE2. Consequently, there is a decrease in the degradation rate of EE2 due to competition for adsorption sites.^9^

The adsorption of EE2 and its by-products within the UiO-66 MOF cavities can be confirmed through XPS analyses conducted on the Nt/Ti-5.0W@UiO-66 electrodes after the degradation process of water supply enriched with EE2 for 30 min (Figure S6). In the high-resolution spectrum measured in the C 1s region (Figure S6a), a peak at approximately 287.5 eV suggests the presence of EE2 adsorbed by the UiO-66 MOF. Additionally, there is an observed increase in the intensity of the C 1s and O 1s peaks compared to the spectra of the same species before the photocatalytic application (Figures 7a,d). Furthermore, a secondary peak observed at approximately 288.0 eV, exceeding the intensity seen in the photocatalyst before its application, can be associated with other organic molecules adsorbed within the adsorbent cavity. This peak likely arises from carbon atoms associated with the O–C=O functional group , indicating that by-products continue to be adsorbed by UiO-66 MOF even after the degradation of the target molecule. This suggests potential readsorption of these intermediate products due to electrostatic compatibility or other affinity mechanisms. The adsorption of EE2 can also be confirmed through the high-resolution O 1s spectrum (Figure S6b), where a peak at approximately 533.0 eV was observed. According to Sydorchuk et al., this peak corresponds to single-bonded oxygen atoms in aromatic groups (O–C=O), which are characteristic bonds present in the EE2 molecule and its by-products, as indicated by the degradation pathway described by de Oliveira et al.^9,54^ Importantly, this type of bond is absent in the composition of UiO-66 MOF. In a study conducted by Jiang et al., which compared the removal efficiency of EE2 using magnesium and ferric oxides, the adsorption of the hormone onto the oxides was confirmed through XPS analysis.^62^ A peak at approximately 533.0 eV in the O 1s spectral region was assigned to the EE2 molecule, indicating its presence on the surface of the oxide materials.

Therefore, the confinement of EE2 and intermediate products within the UiO-66 cavity favors complete degradation of the pollutant, followed by the degradation of these compounds by the OH^·^ radical generated on the surface of the photocatalyst. Similar cooperative effects have been reported in the literature through combinations of photocatalytic and adsorbent materials.^63−66^

Additionally, the toxicity of the initial solution containing 10 mg L^–1^ E1 and EE2 hormones, as well as the final solution from each degradation process with Nt/Ti-5.0W@UiO-66, was assessed by using *S. aureus* and *E. coli*. The toxicity of the final solution after each cycle test was specifically evaluated for EE2. The results are presented in Figure S8 (SI). The initial and final solutions for the two hormones (E1 and EE2) exhibited a low toxicity. However, the intermediated solutions from the degradation experiments showed moderate toxicity, inhibiting the growth of *S. aureus* and *E. coli* by 42% for E1(1 min) and 11% for EE2(15 min). Therefore, the application of Nt/Ti-5.0W@UiO-66 as a photocatalyst for the removal of E1 and EE2 from supply water by a photoelectrocatalytic process is expected to be environmentally friendly after 2 and 30 min of reaction for E1 and EE2, respectively.

## 4 Conclusions

Despite many challenges in applying photoelectrocatalytic treatment to low concentrations of pollutants in large volumes of water, our study demonstrates that nanotubular oxide surfaces grown on Ti*x*W alloy (*x* = 0.5 and 5.0 wt %) modified with UiO-66 MOF may be used as effective photocatalysts to overcome some of these limitations. An increase of around 40% in the degradation of E1 and EE2 in synthetic water for all photocatalysts modified with UiO-66 compared to unmodified ones was observed. This suggests a collaborative effect between Nt/Ti*x*W and UiO-66, minimizing the influence of species diffusion in solution on the treatment efficiency.

In treating a real water matrix, a competition between the target pollutants and other organic compounds occurred. However, the degradation rate was enhanced by up to 1.78 times for E1 and 18.2 times for EE2 compared with the unmodified photocatalyst. These results were reached due to electrostatic attraction and compatibility of the hormones and their intermediates with UiO-66, favoring the adsorption of these compounds from the solution, enhanced by NH_2_ groups linked to the UiO-66 MOF structure, while Ti–O–W nanotubes generated photogenerated OH^·^ radicals essential for hormone degradation.

The Nt/Ti-5.0W modified with a UiO-66 electrode was the most efficient and stable during the recycle tests, proving to be a promising material for applications in photo(electro)catalytic processes for treating water supplies with low pollutant concentrations.

## Supporting Information Available

Physisorption data and BET method, FITR spectrum, distribution of nanoparticles and SEM image, EDX mapping, sorption studies, XPS analysis, XRD data after PEC, toxicologic tests of hormones, and by-products (PDF)
