# Supplementary material for: Coupling UiO-66 MOF with a Nanotubular Oxide Layer Grown on Ti-W Alloy Accelerates the Degradation of Hormones in Real Water Matrices
Source: ACS Omega. 2024 Nov 22;9(49):48571–85. doi: 10.1021/acsomega.4c07470 (PMC11635509; doi:10.1021/acsomega.4c07470)
Supplement: Supplementary file 1 — ao4c07470_si_001.pdf [file ao4c07470_si_001.pdf]

Supplementary Information

Coupling UiO-66 MOF with nanotubular oxide layer grown on TiW alloy accelerates the degradation of hormones in real water matrices

Isabela Disigant[a,b], Juliana de Almeida[a,b], Débora Noma Okamoto[c], Rodnei Bertazzoli[b,d] and Christiane de Arruda Rodrigues*[a,b]

[a]Department of Chemical Engineering, Instituto de Ciências Ambientais, Químicas Farmacêuticas, Universidade Federal de São Paulo, Rua São Nicolau, 210, Diadema, SP, 09913-030, Brazil.

[b]Unesp, National Institute for Alternative Technologies of Detection, Toxicological Evaluation and Removal of Micropollutants and Radioactives (INCT-DATREM), Institute of Chemistry, P.O. Box 355, 14800-900 Araraquara, SP, Brazil.

[c]Department of Pharmaceutical Science, Instituto de Ciências Ambientais, Químicas Farmacêuticas, Universidade Federal de São Paulo, Rua São Nicolau, 210, Diadema, SP, 09913-030, Brazil.

[d]School of Mechanical Engineering, Universidade Estadual de Campinas, Rua Mendeleyev, 200, Campinas, SP 13083-860, Brazil.

***Corresponding author**

Name: Christiane de Arruda Rodrigues

Phone:+55 11 3385-4137 (extension 3538)

Address: Laboratório de Engenharia e Controle Ambiental, Departamento de Engenharia Química, Universidade Federal de São Paulo, São Nicolau St., 210, Diadema, São Paulo State, Brazil, 09913-030.

E-mail address: christiane.arruda@unifesp.br (C. A. RODRIGUES)

Physisorption data and BET method of UiO—66 nanoparticles as-prepared

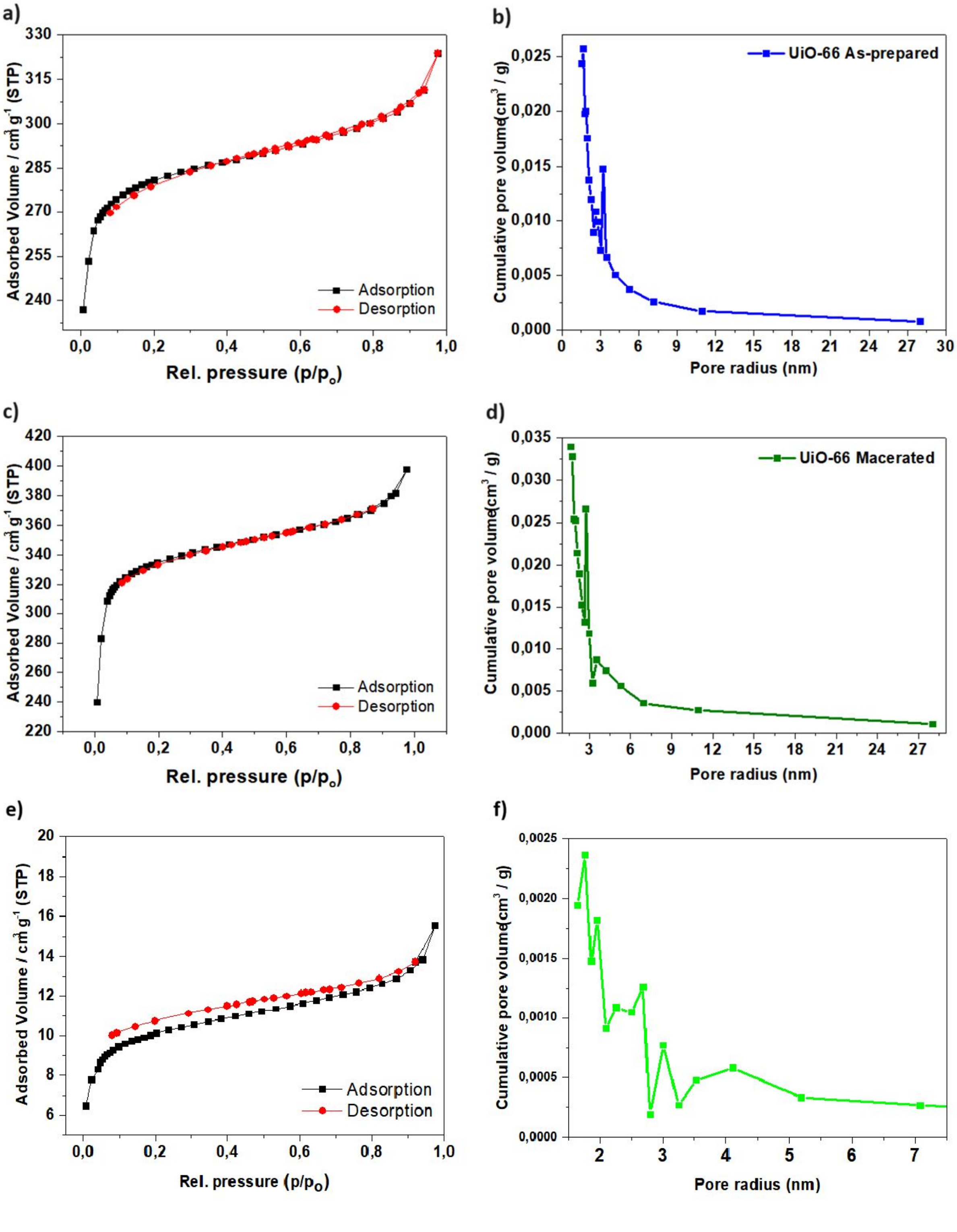

Figure S1. BET analysis with N_2 adsorption-desorption isotherm and BJH method of pore size distribution: a - b) as prepared UiO-66, c - d) UiO-66 after maceration and e - f) UiO-66 after maceration heat treatment 80ºC.

FTIR analysis of UiO-66 nanoparticles

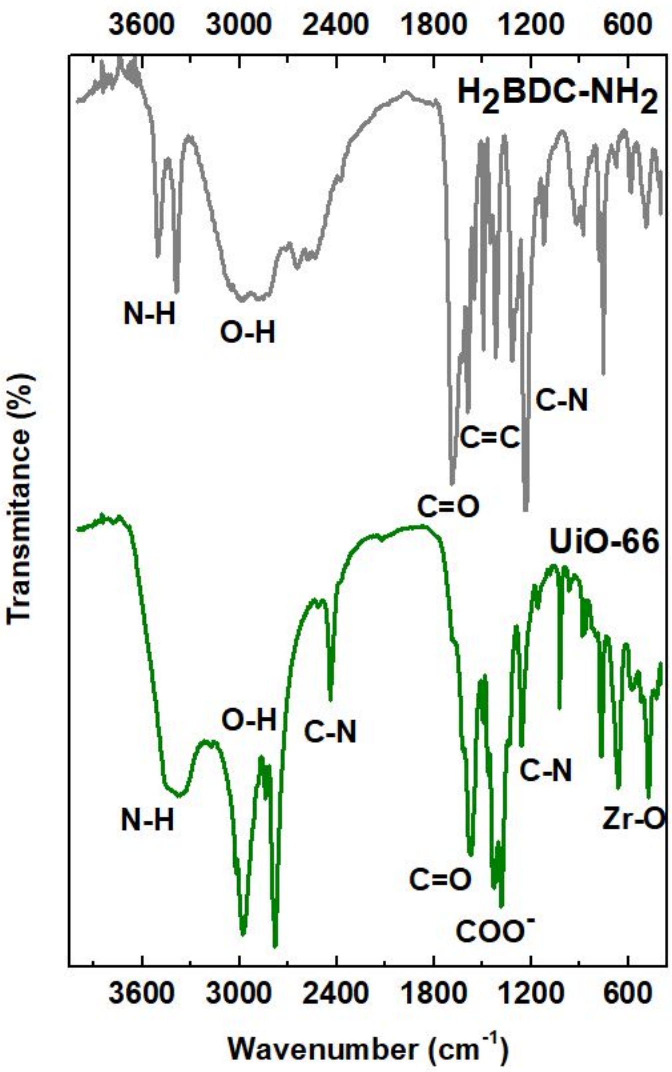

Figure S2. FT-IR spectrum of UiO-66 after the maceration process, compared with the spectrum of 2-Aminoterephthalic acid ($H_2BDC-NH_2$).

UiO-66 nanoparticles distribution size

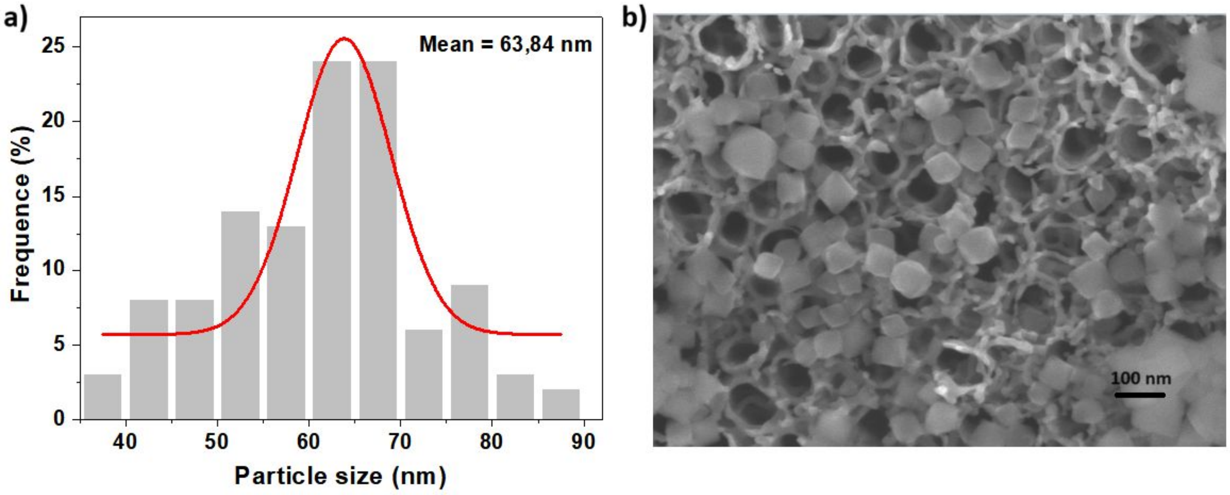

Figure S3. a) Particle size distribution after the maceration process; b) UiO-66 nanoparticle with defined octahedral morphology on Nt/TiO_2.

SEM images and EDX mapping of Nanotube surface with UiO-66 nanoparticles

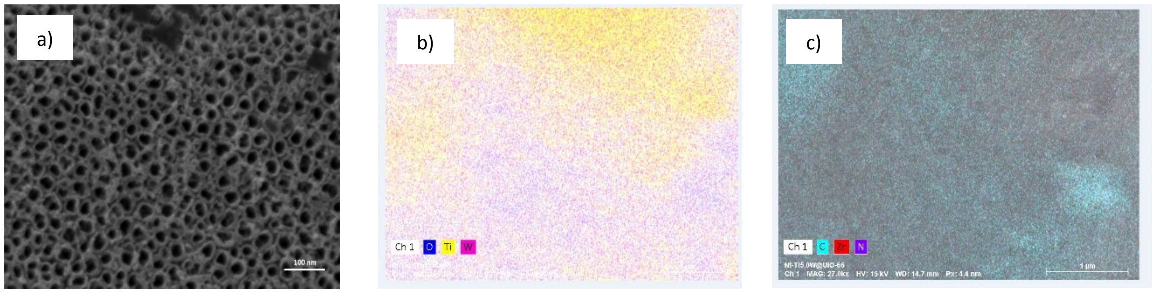

Figure S4. a) SEM image of Nt/Ti5.0W@UiO-66 electrode. b and c) Elemental mapping by SEM/EDX for Nt/Ti5.0W@UiO-66 electrode

Profile of sorption tests for modified nanotubes

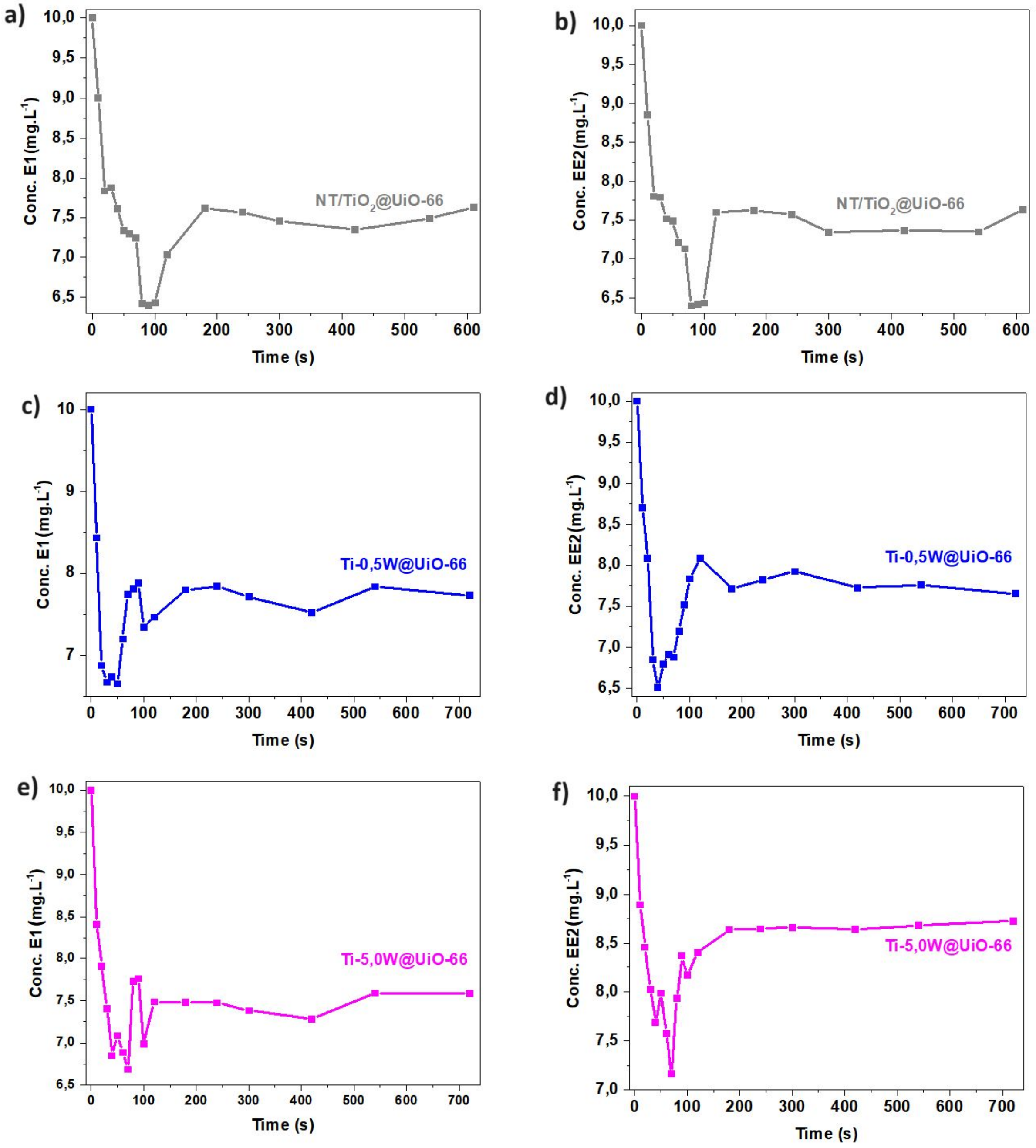

Figure S5. Equilibrium concentration obtained for (a-b) Nt/TiO_2@UiO-66, (c-d) Nt/Ti-0.5W@UiO-66 and (e-f) Nt/Ti-5.0W@UiO-66 by E1 and EE2 (10 mg L^{-1}). Absorption-adsorption and contact time: 12 min.

XPS analyses of photocatalysis after PEC

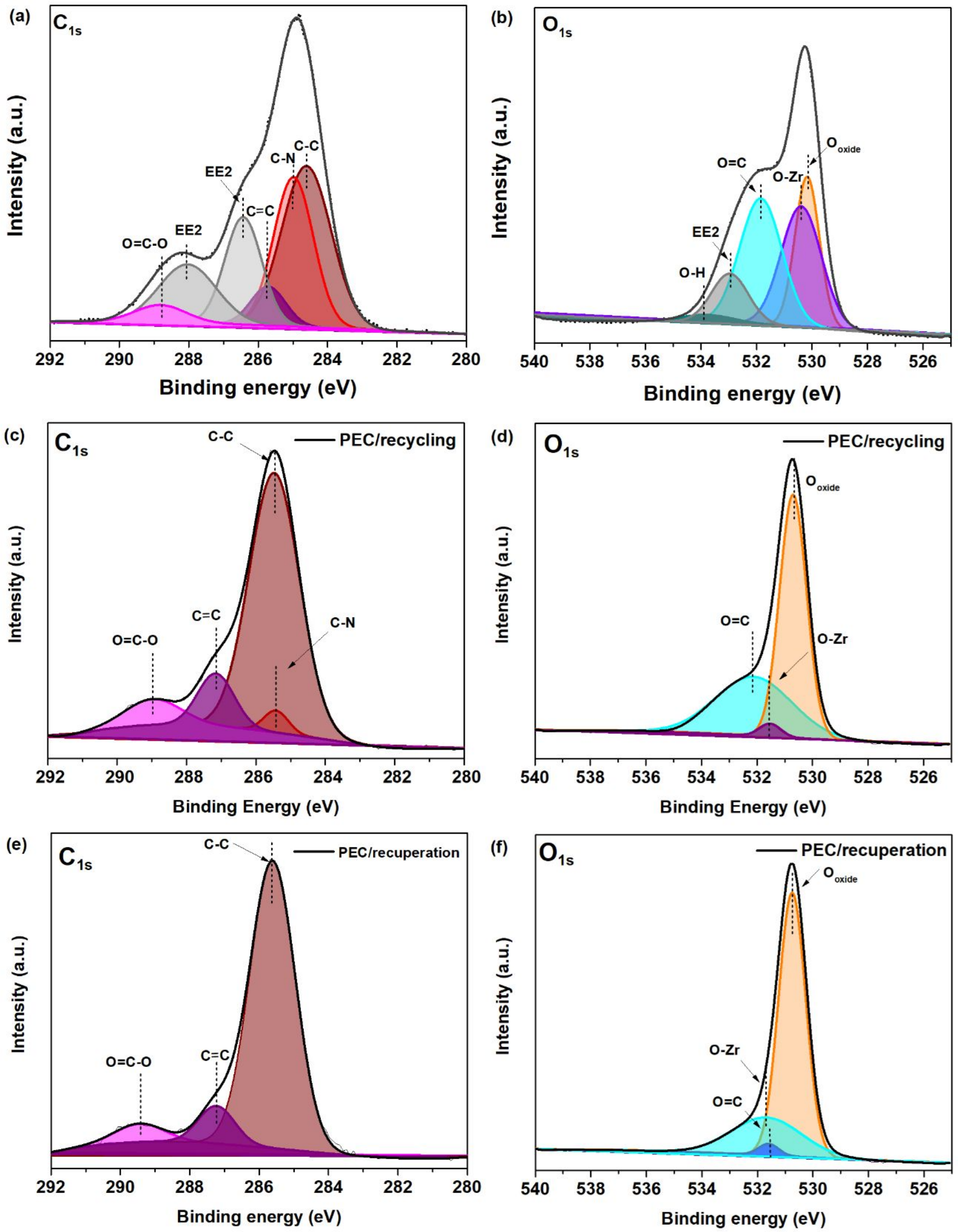

Figure S6. High resolution deconvoluted of C 1s and O 1s spectra from sample of Nt/Ti-5.0W@UiO-66 (a-b) as-prepared and after photoelectrocatalysis application in the EE2 degradation (c-d) after recycling tests and (d-f) after recuperation.

XRD analyses of photocatalyst after PEC and stability tests

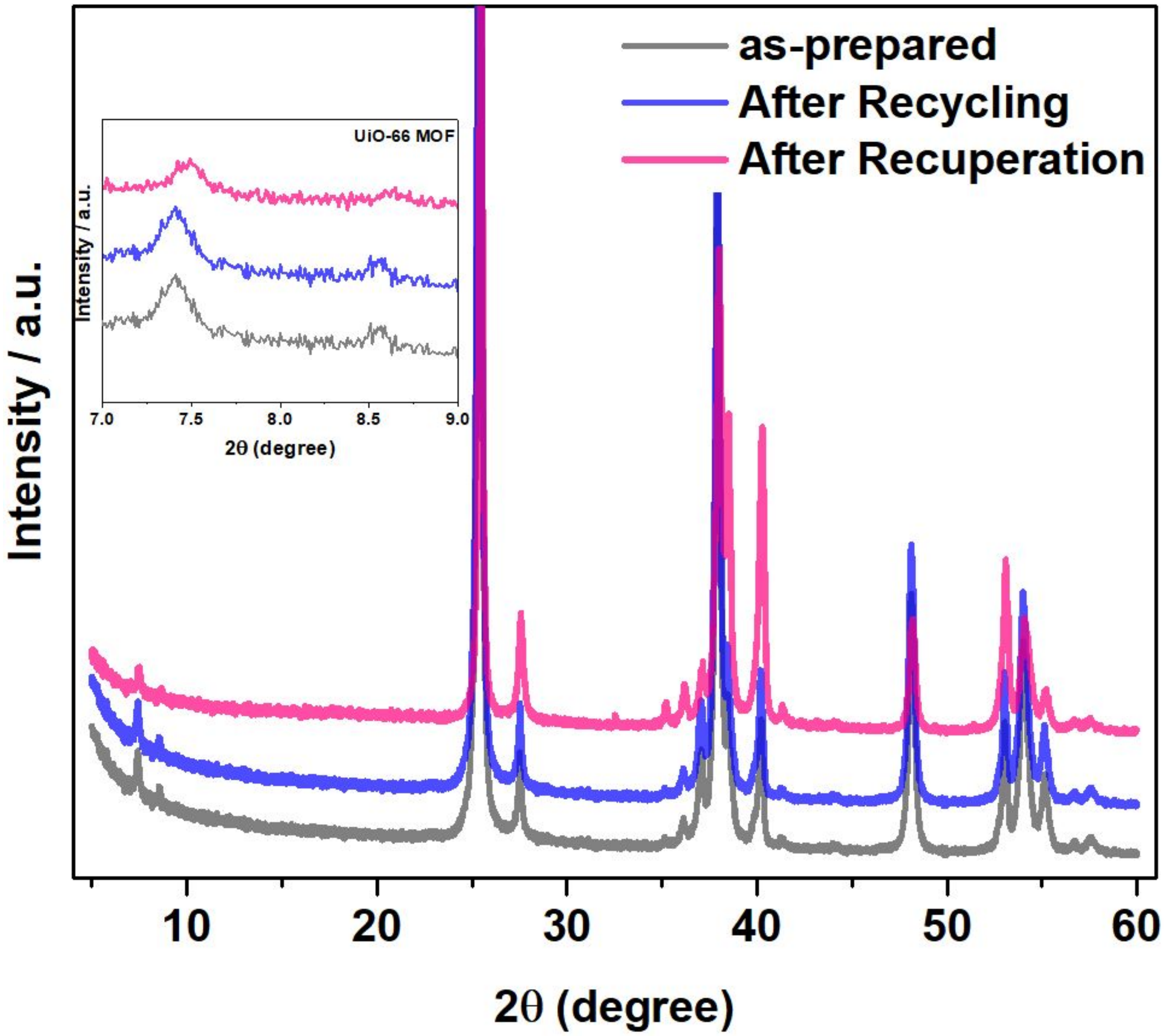

Figure S7. Comparison to XRD analysis from a sample of Nt/Ti-5.0W@UiO-66s as-prepared and after photoelectrocatalysis application in the EE2 degradation after recycling tests and after recuperation using the chronoamperometric method and light irradiation.

Toxicologic test of hormones and intermediated by-products

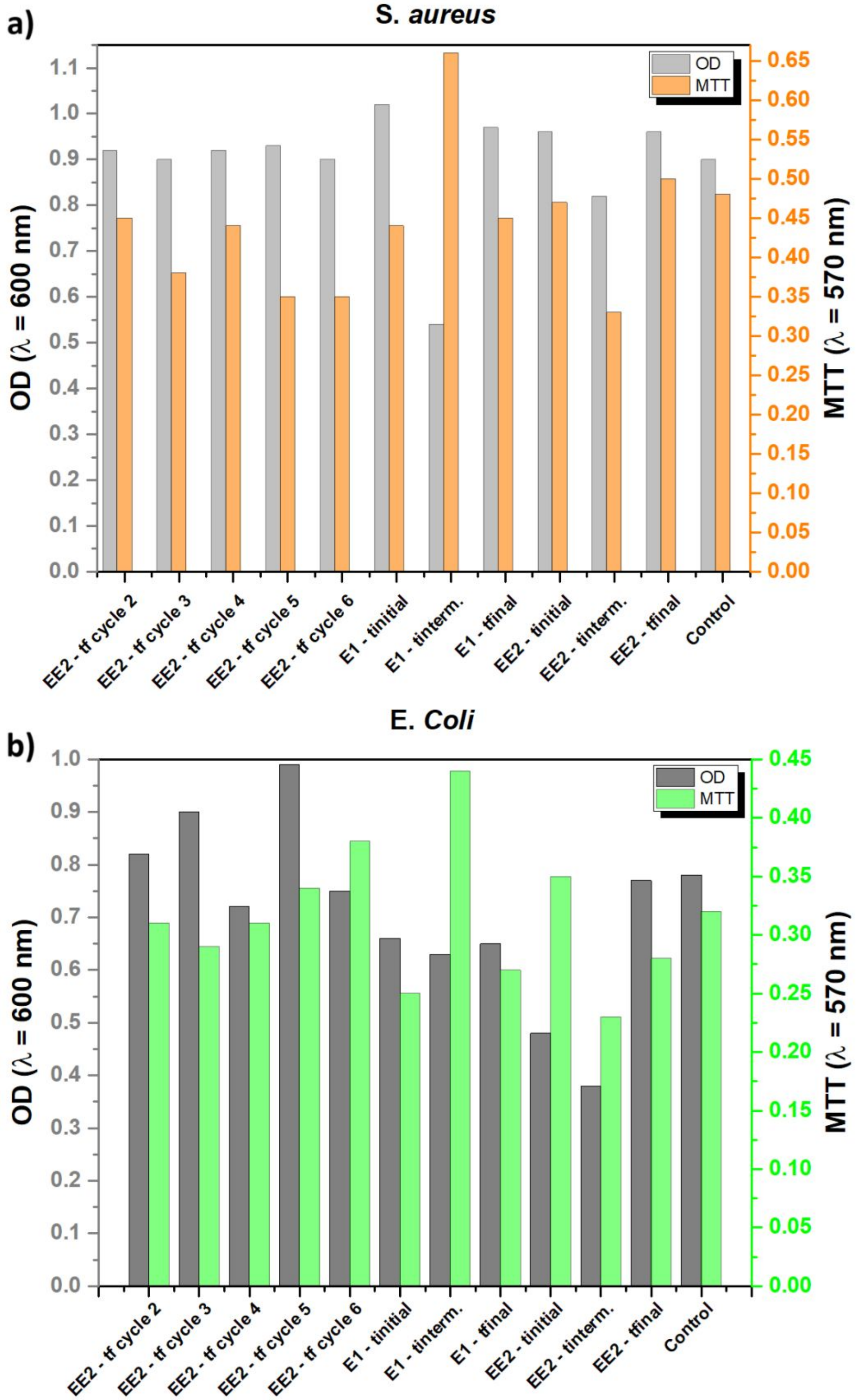

Figure S8. The effect of E1 and EE2 solution in matrix water contained 10 mg L^{-1} before protoelectrocatalytic process, intermediate time and final solution on S. *aureus* biofilm formation. *error bars represent standard deviations.

E1 and EE2 solubility

Table S1. Effect of contact time on the adsorption of the E1 and EE2 hormones over the metal-organic framework UiO-66 as-prepared nanoparticles at pH 6.5 and 7.0 after 3 and 60 minutes.

**Nanoparticles of UiO-66 as prepared on the E1 and EE2**				
	**Conc. E1 (mg L^{-1})**		**Conc. EE2 (mg L^{-1})**	
**Time (min)**	**pH 6.5**	**pH 7.0**	**pH 6.5**	**pH 7.0**
T_0	10.0	10.0	10.0	10.0
T_3	2.6	4.1	7.7	7.3
T_{60}	0.0	0.0	0.0	0.0
